# Revealing Anisotropic Growth of Liraglutide Oligomers by Native Ion Mobility Mass Spectrometry and Molecular Dynamics Simulation

**DOI:** 10.1021/acscentsci.5c00431

**Published:** 2025-06-16

**Authors:** Zhenyu Xi, Syuan-Ting Kuo, Xiao Cong, Xin Yan, David H. Russell

**Affiliations:** † Department of Chemistry, 14736 Texas A&M University, College Station, Texas 77843, United States; ‡ Boehringer Ingelheim, Ridgefield, Connecticut 06877, United States

## Abstract

Oligomerization is an intriguing problem, particularly at the early stages of soluble prefibril oligomer formation, where the growth mechanisms of these species remain difficult to study due to their complexity and heterogeneity. Liraglutide, a widely used drug for diabetes and obesity, has demonstrated various oligomerization outcomes in different studies. In this study, we integrate native ion mobility mass spectrometry (nIM-MS) and molecular dynamics (MD) simulations to unravel the assembly mechanisms of liraglutide oligomers. Our findings reveal that while assembly pathways vary in their steps, they consistently converge into a structure resembling a fuzzy oil drop model. Furthermore, a key residue is identified as a determining factor in oligomerization. The preference for specific shapes varies at different stages of oligomer formation depending on the oligomer size. A theoretical model is proposed to fit collisional cross-section (CCS) data and is verified through both nIM-MS experiments and MD simulations, ultimately establishing an anisotropic growth mechanism for liraglutide. Additional MD simulations provide deeper insights into monomer conformations, which are closely linked to oligomer formation. A bias toward forming extended monomers is shown to facilitate the assembly of larger oligomers in this self-assembling system.

## Graphical abstract

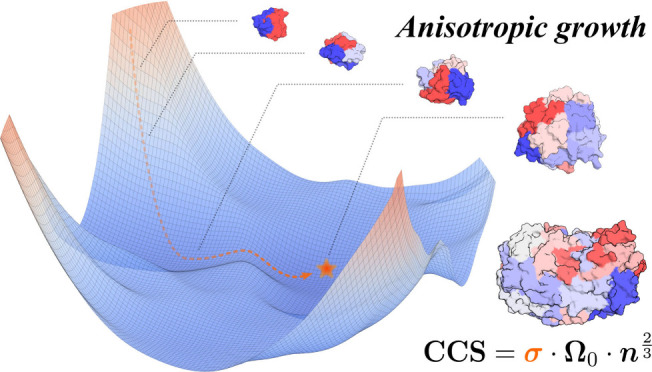

## Introduction

Since its approval by the U.S. Food and Drug Administration in 2010, liraglutide has become a pivotal medication in the management of type 2 diabetes mellitus and obesity. As a glucagon-like peptide-1 (GLP-1) receptor agonist, liraglutide mimics the incretin hormone to enhance insulin secretion, inhibit glucagon release, and delay gastric emptying, thereby improving glycemic control and promoting weight loss.^1^ Although the clinical efficacy of liraglutide is well-documented, its oligomerization behavior remains underexplored. This aspect has rarely been addressed specifically. This lack of focus leaves the oligomeric state shrouded in debate and uncertainty, as studies have reported varying forms such as 5/6-mer,^2^ 6/7/8-mer,^3^ 8/12-mer,^4^ or 7/13-mer.^5^ Despite these ambiguities, the impact of oligomerization on pharmacokinetics, stability, and bioavailability is significant, presenting notable challenges in formulation and therapeutic applications.^6,7^ There is an urgent need to characterize the exact oligomerization states and gain a deeper understanding of the oligomerization dynamics.

The heterogeneity of liraglutide in solution stemming from uncertain oligomerization states complicates the characterization of its behavior in vivo and in pharmaceutical formulations. Traditional analytical methods often fall short in resolving these transient and dynamic oligomeric species. For instance, size exclusion chromatography (SEC) and dynamic light scattering (DLS) provide average molecular weights and size distributions but lack the resolution to distinguish between oligomers with similar sizes or to detect transient intermediates.^8,9^ Nuclear magnetic resonance (NMR) spectroscopy and X-ray crystallography offer detailed structural insights but are limited by sample requirements and difficulties in analyzing heterogeneous complexes or transient assemblies.^10,11^

Native mass spectrometry (nMS) offers significant advantages in this context, enabling the characterization of biomolecules in their native or near-native states while providing detailed information on their mass, charge, and conformation.^12–17^ A number of nMS studies have demonstrated robustness and versatility, including determining stoichiometry and thermodynamics from protein aggregation to ligand binding.^18–21^ Furthermore, nMS coupled with ion mobility spectrometry (IMS), known as native ion mobility mass spectrometry (nIM-MS), provides an additional dimension by separating ions based on their shape and charge, allowing for the investigation of biomolecular conformations and their structural heterogeneity.^22–24^ For instance, IM-MS has been utilized to elucidate the structural dynamics of protein aggregation and to study protein conformational changes involved in folding and misfolding processes.^25–28^

To complement experimental observations, molecular dynamics (MD) simulations can provide more atomic details of oligomerization processes. However, conventional all-atom MD simulations are limited by computational cost, especially when simulating large systems over long time scales that are necessary to capture the complete oligomerization pathways.^29^ Existing oligomerization studies rely on assumptions such as manual monomer placement and exhaustive searches.^3^ To overcome these challenges, coarse-grained (CG) simulations provide a practical alternative by simplifying the system, enabling the exploration of extended time scales and allowing oligomerization to be simulated as a self-assembly process.^30^ Additionally, advancements in backmapping techniques have made it possible to retrieve detailed atomic-level information from CG simulations, effectively bridging the gap between residue-level resolution and atomic-resolution structures.^31^

The integration of nIM-MS data with MD simulations significantly enhances the accuracy and reliability of the conformational models. This combined approach has been instrumental in elucidating protein complex dynamics, offering insights unattainable through either method alone.^32–34^ Additionally, collisional cross-section (CCS) trendline analyses from nIM-MS can provide valuable mechanistic details, aiding in the interpretation of oligomerization pathways.^35,36^ In this study, we introduce an anisotropic model to investigate the growth mechanisms of liraglutide oligomers. Leveraging nIM-MS and advanced MD simulation techniques, we have successfully uncovered the structural dynamics and mechanism of liraglutide oligomerization.

## Methods

### Native Ion Mobility Mass Spectrometry

Ion mobility mass spectrometry (IM-MS) experiments were performed by using a Synapt G2 traveling wave ion mobility mass spectrometer (Waters Corp., Milford, MA, U.S.). Liraglutide (98.91% purity) was obtained from MedChemExpress LLC (Monmouth Junction, NJ, U.S.). The sequence is “HAEGT FTSDV SSYLE GQAAK EFIAW LVRGR G”, where Lys20 (using 1-based indexing) is covalently linked to a *N*-hexadecanoyl-l-glutamic acid group. This modified residue is termed D6M, in alignment with PDB notation and for simplification. The liraglutide powder was dissolved to a final concentration of 1 mg·mL^–1^ in 20 mM ammonium acetate (AmAc) at pH 6.7. Freshly prepared samples were analyzed immediately to capture small oligomers (*n* = 2–7) under the specified solution conditions. For the analysis of larger oligomers (*n* > 7), the sample was incubated at 37 °C for 96 h to allow their formation.

The prepared sample was introduced via a nanospray electrospray ionization (nano-ESI) source using gold-coated pulled borosilicate capillaries. The spray voltage was maintained between 1.3 and 1.8 kV. To minimize gas-phase activation of the liraglutide oligomers, activation parameters were carefully examined. Key instrumental parameters included a source temperature of 50 °C, sampling cone voltage of 20 V, extraction cone voltage of 0.3 V, trapping gas flow rate of 8 mL·min^–1^, and trap collision energy of 4 V. The transfer collision energy was set to 0 V for small oligomers (*n* < 12) and 50 V for larger oligomers (*n* > 12). This increase in collision energy after ion mobility separation aimed to enhance signal clarity within the *m*/*z* range of 5000–6000 without significantly altering analyte drift times (see the Supporting Information (SI)). Furthermore, premobility mass selection was implemented before ion mobility measurement to minimize interference from other gas-phase fragment ions (see the SI for instrument configuration). Collision cross-sections were calibrated using multiple charge states of denatured myoglobin, cytochrome c, and ubiquitin.^12^ Raw MS and CCS data are provided in SI section S1.

### Molecular Dynamics Simulation

To obtain reliable oligomer structures with minimal human bias, no predefined positioning, rotation, or placement of peptide monomers was applied. Instead, peptides were randomly distributed at the beginning of the simulation, and the assembly process was monitored over time. A substantial number of solvent molecules were included to approximate the experimental peptide concentrations and allow for realistic aggregation behavior. In contrast, conventional simulations typically use a minimal solvent environment (e.g., a water box extending 10 Å from the protein surface), which would be insufficient for accurately modeling oligomerization in this context and could potentially introduce artifacts.

Simulating large systems, however, is computationally demanding even on state-of-the-art GPU clusters, especially for those containing a large number of solvent molecules. To overcome this limitation, we employed a coarse-grained (CG) approach using the MARTINI force field,^37,38^ which significantly reduces computational cost by representing defined chemical moieties as beads. Each bead is assigned predefined properties, such as mass, charge, and Lennard-Jones parameters, allowing for a simplified yet effective representation of molecular interactions.

Using this approach, a test run of the heptamer assembly simulation demonstrated a substantial performance gain. The MARTINI-based CG system, consisting of 31,045 beads, achieved a simulation speed of 14,477.9 ns/day. In contrast, the equivalent all-atom simulation using the CHARMM36m force field, involving 379,773 atoms, ran at only 122.1 ns/day on an NVIDIA RTX 4090 GPU paired with an Intel i9-12900K CPU. Therefore, while atomic-level simulations at the microsecond scale could take almost 100 days to complete, the CG approach enables comparable time scales to be reached within a single day.

To further accelerate the CG simulation, we implemented simulated annealing, a technique that periodically and gradually increases and then decreases the system temperature. This approach helps the system escape local energy minima and promotes sampling of a broader conformational space. As a result, it facilitates the removal of suboptimal contacts and enables the structure to overcome energy barriers, allowing it to rapidly converge toward a lower-energy conformation. This comes at the cost of losing detailed kinetic information, as the accelerated temperature transitions do not reflect the natural time scale of molecular events.

One limitation of the CG method is that the chemical detail is inherently reduced. The bead representation lacks the explicit atomic resolution necessary for interpreting fine structural and chemical interactions. Fortunately, tools such as backward.py have been developed to backmap coarse-grained beads into full atomic structures.^31^ This enables researchers to recover atomistic representations from CG trajectories, allowing detailed structural analyses without sacrificing chemical information.

The CG approach enables us to convert CG solution-phase structures into atomistic representations, allowing for the protonation of titratable residues or chemical moieties, such as N- and C-termini. Only with this step is it feasible to prepare simulation input structures that accurately reflect specific protonation conditions in the gas phase. With these representative solution-phase structures extracted and converted to atomistic resolution, they were used as starting points for gas-phase atomistic simulations. GROMACS^39^ with the force field CHARMM36m^40^ implemented was used for these simulations.

The total charge of each ion was set to match the most abundant charge state observed experimentally. Protonation sites were assigned automatically following this protocol: (a) Preservation of existing positive charges: protons on positively charged sites (e.g., Lys, Arg, His, N-termini) were retained as in the solution-phase structure. (b) Protonation of accessible acidic sites: negatively charged, solvent-accessible sites not involved in salt bridges were protonated next. Protonation order was determined by descending p*K*_a_, followed by descending SASA. (c) Salt bridge-associated acidic sites: negatively charged, solvent-accessible sites involved in weaker salt bridges were protonated afterward, prioritized by ascending salt bridge distance, then descending p*K*_a_, and finally descending SASA. (d) Buried acidic sites: negatively charged, solvent-inaccessible sites were considered for protonation last. However, this final step was never reached in the simulations of the most abundant charge states.

The gas-phase structures, with properly assigned protonation sites, were allowed to relax for 10 ns. This time scale is intentionally conservative, as most structures reached equilibrium within the first 5 ns. The final 5 ns of each trajectory were used for subsequent CCS calculations using both IMPACT^41^ and PSA.^42–46^ Shape factor calculation was based on MSMS.^47^

As for the free monomer simulation, we also examined the monomer on the microsecond time scale. Unlike the oligomerization simulations, which aim to rapidly identify equilibrated assembly structures, the primary objective here was to sample a broad range of monomer conformations while preserving a statistically rigorous ensemble. Specifically, we aimed to avoid oversampling unstable high-energy states while also ensuring that rare but physically relevant conformations were not missed.

To achieve this, we employed Replica Exchange Molecular Dynamics (REMD). In this method, multiple replicas of the system are simulated in parallel at different temperatures. Periodic exchanges of configurations between replicas are attempted based on a Metropolis criterion, which allows the system to overcome energy barriers more efficiently and explore a wider conformational space.^48,49^ High-temperature replicas enhance conformational transitions, while low-temperature replicas ensure accurate ensemble statistics. As a result, REMD enables the efficient and balanced sampling of both dominant and minor conformational states. The structures collected from the low-temperature replicas were subsequently analyzed to characterize the conformational clusters of the monomer in solution.

Simulation details and setup can be found in SI section S2.

### Anisotropic Growth Model

The CCS value reflects the apparent size of an object by representing the averaged projected area across all orientations.^50^ When structural information (PDB files) about a protein is available, CCS can also provide insights into its overall shape, such as whether it is more prolate or oblate.^51^ However, without prior knowledge, the specific shape cannot be uniquely determined from CCS alone. Previous studies have shown that a series of CCS values across different oligomeric states can be fitted with trendlines to infer general shape characteristics.^12,36^ Despite this, the physical meaning of the coefficients derived from these trendlines remains unclear. In this study, we provide a mathematical interpretation of the coefficient associated with the isotropic model (Figure 1A). Rather than being treated as a purely empirical or negligible fitting parameter, this coefficient is demonstrated to represent a distortion factor, quantifying the deviation of an object from a perfectly spherical shape. With this clarified interpretation, the original isotropic model can be extended and redefined as an anisotropic model. This refined understanding enables its application to a broader range of assemblies with more diverse geometries.

**Figure 1:**
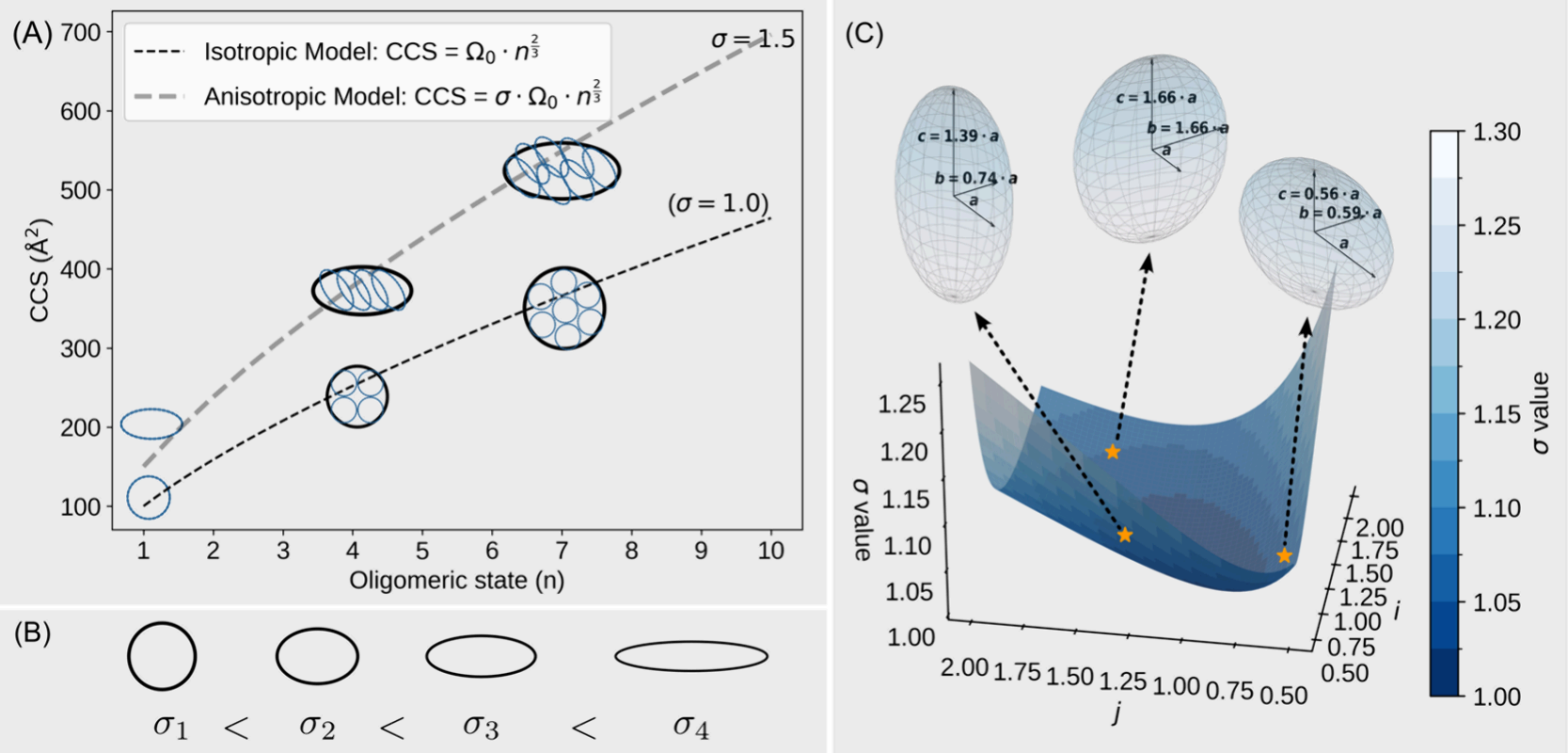
Conceptual representation of the anisotropic growth model. (A) Aggregates composed of ***n*** subunits, shown under isotropic and anisotropic assembly modes. (B) Ellipsoids corresponding to different **σ** values; a higher **σ** indicates greater deviation from a spherical shape. (C) Distortion factor **σ** plotted as a function of ***i*** and ***j***; yellow stars mark points where **σ** equals 1.05. Multiple ellipsoidal configurations can correspond to the same **σ** value.

To support this interpretation, we base our analysis on three key assumptions. First, the mobility of the aggregates is derived from the rotationally averaged aspects of the object, with all orientations equally likely; this implies that the CCS represents the rotationally averaged shadow areas. Second, the aggregates form in a densely packed manner, minimizing the number of voids within the structure. Third, the overall shape of the object is assumed to be perfectly convex. With these assumptions, two equations can be derived as below (stepwise derivation is provided in SI Appendix I).

For an aggregate composed of ***n*** number of monomeric subunits, the CCS follows the relationship

$$\mathrm{CCS}=\sigma \Omega_{0}n^{2/3}$$ 1

where **σ** is the distortion factor and Ω_0_ is the CCS of a single monomeric unit.

A more extreme value of **σ** represents a more distorted ellipsoid from a normal sphere (Figure 1B). As **σ** approaches 1, the assembly is more spherical. Assuming each subunit resembles an ellipsoid, with its semi-axes being ***i*_0_**-fold and ***j*_0_**-fold of its shortest axis and the assembled structure having folds of ***i*** and ***j***, the distortion factor **σ** can be further expressed as

$$\sigma ={\left(\frac{i_{0}j_{0}}{ij}\right)}^{2/3}\frac{{\left(i^{p}+j^{p}+i^{p}j^{p}\right)}^{1/p}}{{\left(i_{0}^{p}+j_{0}^{p}+i_{0}^{p}j_{0}^{p}\right)}^{1/p}}$$ 2

where ***p*** = 1.6075, according to the Knud Thomsen approximation for ellipsoids.

By plotting **σ** as a function of ***i*** and ***j*** and assuming that ***i*_0_** and ***j*_0_** are 1, we can easily know that it is not a monotonic function of either variable. By taking the derivative, we can also easily know it has a local minimum at ***i*** = ***j*** = 1, meaning a sphere has the least CCS compared to ellipsoids whenever the small ellipsoids have assembled. With a fixed value of **σ**, the distortion factor could have multiple solutions with various combinations of ***i*** and ***j*** values, i.e., the ellipsoid could have different semi-axes combination (Figure 1C). **σ** values are also related to eccentricity; a table of comparisons is provided in the SI (Table S4).

### Fuzzy Oil Drop Model

The fuzzy oil drop model describes the organization of a protein, wherein hydrophobic residues migrate toward the core of the complex, shielding themselves from the aqueous surroundings, while a polar interface forms at the surface.^52^ This conceptual framework, modeled by using a three-dimensional Gaussian distribution, is analogous to the structural organization seen in micelles (as a 3D example) and membrane bilayers (as a 2D example). By quantifying the distribution of hydrophobic and hydrophilic residues, the fuzzy oil drop model provides insights into the variable stability of proteins and their associated biological functions.^53^ In this study, we adapt the model to a simplified one-dimensional representation to visualize and analyze directional hydrophobic interactions.

Residue hydrophobicity values *H*(*r*) are normalized by the maximum absolute value from the Kyte–Doolittle scale:^54^

$$H\left(r\right)=\frac{h\left(r\right)}{\max\left(|h|\right)}$$ 3

where *h*(*r*) is the hydrophobicity of residue *r* and max­(| *h* |) is the largest absolute value in the scale.

The spatial spread σ_*r*,*i*_ of each residue *r* was calculated as the root-mean-square deviation (RMSD) of the positions of all its atoms from the residue’s center. For each axis *i*, the spread σ_*r*,*i*_ is defined as

$$\sigma_{r,i}=\sqrt{\frac{1}{N_{r}}\overset{N_{r}}{\underset{j=1}{\sum }}{\left(x_{j,i}-\mu_{r,i}\right)}^{2}}$$ 4

where *x*_*j*,*i*_ is the position of the *j*th atom in residue *r* along axis *i*, μ_*r*,*i*_ is the mean position of the residue *r* along axis *i*, and *N*_*r*_ is the number of atoms in the residue.

The hydrophobicity *H*_*i*_(*x*) at position *x* along axis *i* is calculated by summing the Gaussian-modeled contributions from all residues:

$$H_{i}\left(x\right)=\overset{n}{\underset{r=1}{\sum }}\left[H\left(r\right)\;\exp\left(-\frac{{\left(x-\mu_{r,i}\right)}^{2}}{2{\sigma_{r,i}}^{2}}\right)\right]$$ 5

where *H*(*r*) is the hydrophobicity of residue *r*, μ_*r*,*i*_ is the mean position of the residue, and σ_*r*,*i*_ represents the spatial spread of the residues.

A detailed explanation and code implementation of this fuzzy oil drop model can be found in SI Appendix II.

## Results and Discussion

### Qualitative Overview of the 14-mer Assembly Process

In one of three simulated annealing simulations, snapshots of the simulation were taken and converted to atomistic representations (Figure 2A). The radius of gyration (Rg) calculated from all single peptides was plotted over time (Figure 2B). This Rg value is closely related to the oligomeric state. Each drop in the monitoring curve represents the formation of specific oligomers. At 90 ns, we observed the formation of a dimer and a loosely associated tetramer. By 300 ns, a monomer joined the tetramer to form a pentamer, while additional dimers appeared. At 380 ns, a heptamer was formed, and at 580 ns, the heptamer rearranged into a sphere-like structure. By 730 ns, a dimer and a trimer associated to form a pentamer. At 820 ns, the pentamer further oligomerized to form another heptamer; consequently, the two heptamers existed at a notable distance from each other. Since heating began at 700 ns, it accelerated molecular motion and increased the frequency of molecular collisions. As a result, by 1000 ns, the two heptamers converged to form a dimer of heptamers. As the simulated annealing progressed, the structure was compacted further, transitioning from a prolate-like intermediate to a more sphere-like conformation, as shown in Figure 2A8,A10.

**Figure 2:**
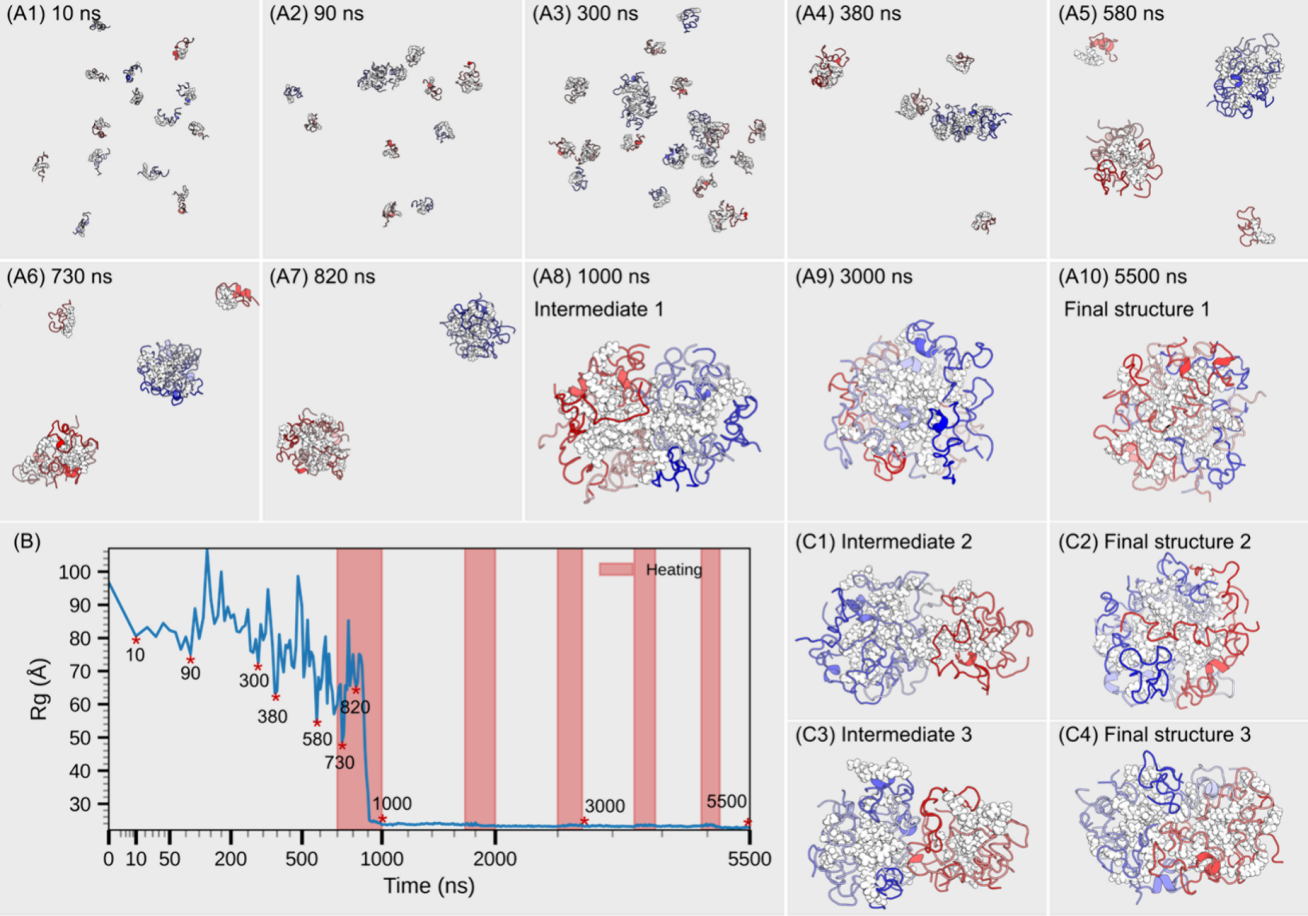
(A) Simulation structures of 14 liraglutide molecules at various time points with each individual peptide colored differently. The side chain atoms of D6M are represented as white spheres. (B) Monitoring of liraglutide assembly via the overall radius of gyration over time, with periodic simulated annealing applied; time axis is warped for clarity, and heating regions are highlighted in red. (C) Representative structures from two additional trajectories.

In the other two replicates of simulations, one 14-mer formed from a 4-mer and a 10-mer, while the other was assembled from a 6-mer and an 8-mer. However, the final structures exhibited substantial structural similarity, as all the side chains of D6M residues were pointed toward the inner center of the whole oligomer (Figure 2C). The only minor difference found was from the third final structure (Figure 2C4). It remained prolate-like even after 5500 ns of simulation, which may require additional annealing cycles to evolve into a compact form. This variation may also be due to some suboptimal contacts and orientations among monomers. For the other two simulations, time-stamped structures and monitoring of Rg over time are provided in the SI (section S3). Similarly, simulations of 2-mer to 7-mer and 30-mer have also been conducted. Representative structures are provided in Figure 5B.

### Quantitative Analysis of Oligomerization

To better visualize the conformational dynamics of oligomerization, principal component analysis (PCA) was applied to reduce the dimensionality of the simulation trajectories, yielding a two-dimensional representation of the conformational landscape. The analysis revealed that, despite being started in clearly distinct conformational spaces, resulting from the initial stochastic placement of monomers, the simulation replicates ultimately converged toward a similar structural ensemble (Figure 2A10,C2,C4).

The corresponding free energy landscape (Figure 3A) illustrates that oligomerization proceeds through multiple assembly pathways, converging into a closely related low-energy basin. In Path I, monomers assemble along a relatively smooth trajectory. The energy landscape does not reveal any pronounced plateau, and the system rapidly descends into the deepest free energy well (denoted as **δ**), with a Δ*G* of −23.01 kcal/mol. As illustrated in the trajectory frames (Figure 2A), this assembly mode corresponds to the fusion of two 7-mers. In Path II, a 4-mer and a 10-mer merge to form a 14-mer (Figure 2C1,C2). This pathway is characterized by an extended plateau region on the free energy surface (denoted as **β**), with a Δ*G* of −22.04 kcal/mol. After residing in this metastable state for approximately 1000 ns, the system overcomes an energy barrier and ultimately transitions into the same deep well **δ** as in Path I.

**Figure 3:**
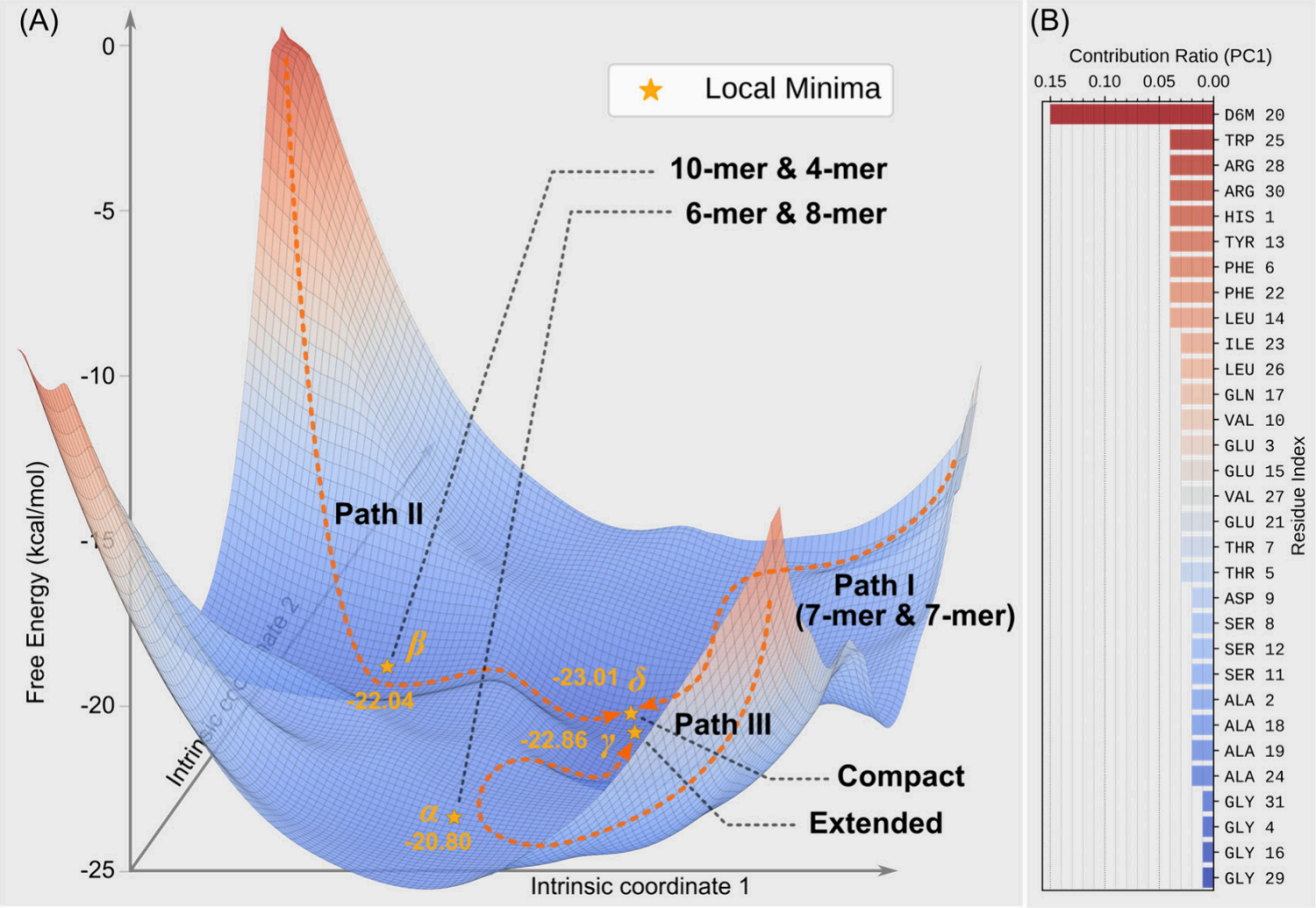
(A) Free energy landscape for 14-mer formation with illustrative arrows indicating distinct oligomerization pathways. The XY coordinates represent dimensionless intrinsic coordinates describing conformational differences. (B) Residue-wise contributions to the assembly process.

In contrast to the first two pathways, Path III follows a markedly different route. In this case, a 14-mer forms from a 6-mer and an 8-mer (Figure 2C3), residing in a separate plateau region (denoted as **α**) with a Δ*G* of −20.80 kcal/mol. Due to suboptimal intersubunit contacts, this pathway fails to form a compact 14-mer and instead produces an extended conformation (Figure 2C4). Consequently, the system settles into a secondary low-energy well (denoted as **γ**) with a Δ*G* of −22.86 kcal/mol. Although this final state is energetically similar to those of Paths I and II, it is structurally distinct. Detailed structural snapshots and temporal evolution of the two paths are provided in SI Figures S5 and S6, respectively.

The free energy landscape construction and identification of local minima (**α**, **β**, **γ**, and **δ**) followed a statistically grounded methodology, as detailed in SI Appendix II. The appendix also includes key scripts and descriptions of statistical procedures. Briefly, the energy values were estimated from population densities using the Boltzmann distribution. Unlike conventional methods that rely on histogram-based binning, which can be highly sensitive to bin size selection,^55^ our approach provides a more stable estimation of free energy values. To overcome this limitation, we adopted an approach that integrates adaptive kernel density estimation with total variation regularization and bootstrap-based uncertainty quantification. This method transforms noisy and unevenly sampled trajectories into a robust and interpretable energy landscape, enabling the reliable identification of metastable states and transition features, even under conditions of data sparsity or imbalance.

A minor concern regarding the constructed free energy surface is that it is based on annealing simulations. As a result, the kinetic aspects of the system may not be fully captured, potentially underestimating the true difficulty of the structural transitions. Since annealing cycles are applied periodically, energetic barriers cannot be reliably quantified. A more rigorous characterization would require input from constant-temperature simulations or enhanced sampling techniques, such as replica exchange molecular dynamics or metadynamics. However, these approaches are computationally demanding and require a delicate setup to avoid kinetic trapping in local minima.

All observations from the simulation led to a critical question: what drives liraglutide to oligomerize? Despite following various assembly mechanisms, the oligomers ultimately converge into highly similar structures.

To obtain residue-level quantification of contributions to oligomerization, we further examined and developed methods to dissect the principal eigenvectors. By analyzing residue-specific motions along these eigenvectors, we quantified each residue’s contribution to the oligomerization process. Notably, the engineered residue D6M exhibited the highest contribution, accounting for 15.1%. This finding also corroborates observations made during the oligomerization process, where the lipid tail appears to function as a “searching guide.” It rambles along the surface of oligomers, seeking an appropriate position and angle on the oligomers and facilitating assembly into larger ones (Figure 2A6 to A7). The tail can also swing or diffuse inward to maximize hydrophobic contacts, aiding the fusion of two smaller oligomers into a larger one (Figure 2C1 to C2). Moreover, all charged residues collectively contribute significantly to oligomerization, accounting for 23.9% (Figure 3B), signifying the role of Coulombic interactions in maintaining the overall structure, as salt bridges have been frequently identified on the surfaces of the oligomers. These findings indicate that the hydrophobic effect together with Coulombic interactions are the key factors driving oligomerization in the liraglutide system.

Besides that, these peptides assemble into a micelle-like system, where the fatty tail converges into the core of the assembly. To quantify this effect and assess its stability, the fuzzy oil drop model was applied (Figure 4). As a general observation, the hydrophobicity profiles along the *x*-, *y*-, and *z*-directions consistently show negative values at the edges (normalized positions around −0.75 and +0.75), indicating that the surfaces of all three path products are predominantly hydrophilic. When focusing on the average hydrophobicity across all three directions, the product of Path I exhibits a pronounced peak, particularly along the *y*-axis, suggesting a well-defined hydrophobic core. In contrast, the Path III product displays a relatively flat hydrophobicity profile. Markedly, in the *z*-direction, it shows a central dip flanked by two shallow peaks, implying the presence of internal hydrophilic interactions. These internal features likely hinder the system from forming a more compact aggregate. Further analysis of the internal hydrophilic regions from the Path III product reveals key polar interactions, including a salt bridge between H1 and E3 as well as a hydrogen bond between T5 and E21. The overall similarity in hydrophobicity profiles between the Path I and Path II products suggests structural resemblance, whereas the divergent profile of Path III points to a different structural organization.

**Figure 4:**
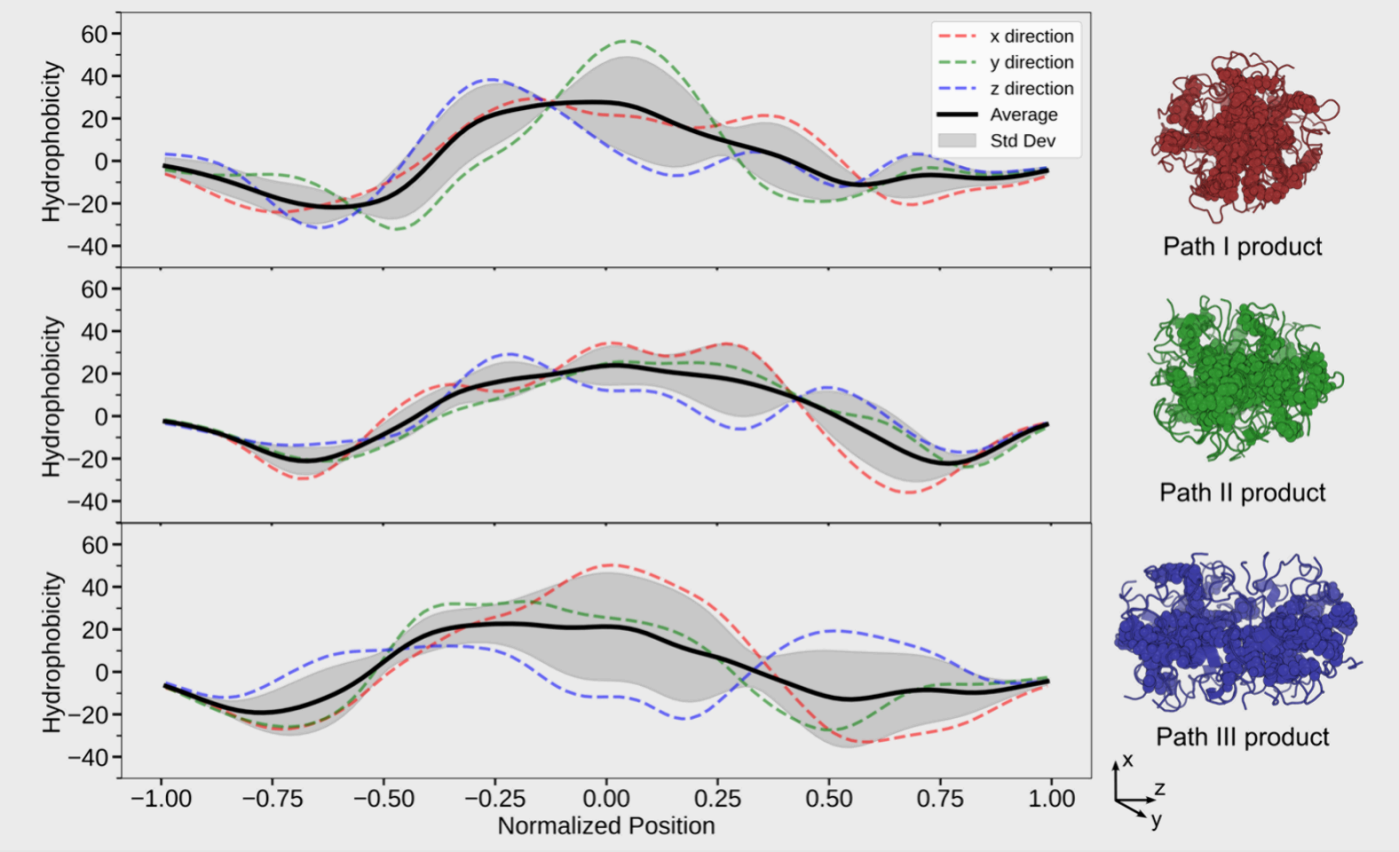
Oligomer structures interpreted using the fuzzy oil drop model, with three curves representing hydrophobicity along the *x*-, *y*-, and *z*-directions, as well as the average across all three. Corresponding 14-mer structures are shown on the right with fatty tail chains highlighted as spheres and peptide backbones represented as lines.

### CCS Trendline Analysis

To validate the reliability of the simulation results, nIM-MS was used to examine oligomer structures and provide a conformational profile in terms of CCS. In Figure 5, experimental CCS values are represented as circles with their sizes scaled logarithmically according to the relative peak intensities of the corresponding charge state. It should be emphasized that although some conformations may appear visually larger in the plot, their actual intensities are much lower. The use of a logarithmic scale is intended to enhance the visibility of low-abundance species that would otherwise be difficult to distinguish on a linear scale (see the detailed CCS list in SI Table S1).

**Figure 5:**
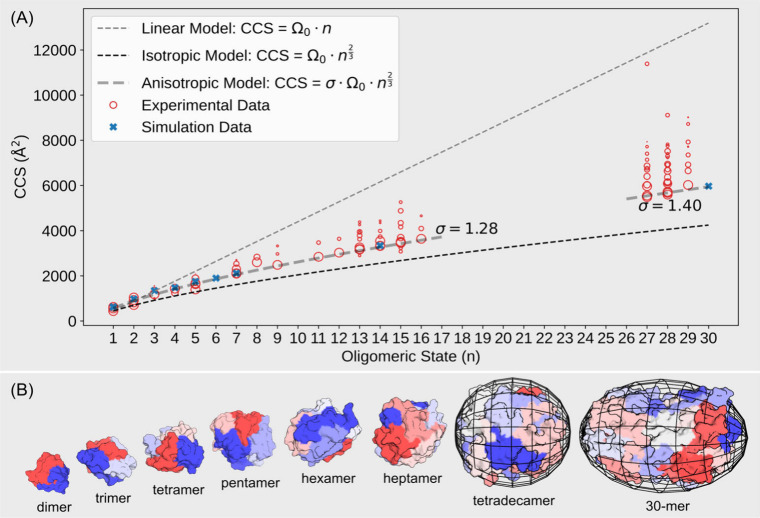
(A) Linear, isotropic, and anisotropic models used to fit the growth patterns of oligomers. Circle sizes are log-proportional to conformation abundances. (B) Surface-style representation of simulated oligomers, with each peptide colored differently. The 14-mer and 30-mer structures are enclosed within ellipsoids that best accommodate them.

It is important to note that a single oligomer can exist in multiple charge states and each charge state may adopt several distinct conformational states. This size-scaled representation provides a clear visualization of the conformational diversity: more abundant states appear as larger circles, while smaller circles often represent less populated, possibly intermediate structures or minor products. In some cases, particularly for the ∼14-mer and ∼28-mer, these species exhibit trailing distributions: a series of smaller circles extend beyond the main population, which may reflect minor species or suboptimal assembly intermediates. This is also supported by our simulations, where a particular 14-mer was formed with two distinct cores and failed to reorganize into a single core micelle-like structure, even after 5500 ns of simulation time.

Monomer conformational heterogeneity is clearly evident in the nIM-MS data, as shown by the large, well-separated circles at *n* = 1. Distinct monomer conformations were observed with a CCS of 439.7 Å^2^ for the [1]^2+^ species and CCS values of 558.8 and 627.3 Å^2+^ for the [1]^3+^ species, all exhibiting considerable signal intensity. This heterogeneity highlights the structural diversity of monomers and motivates further simulation analyses focused specifically on monomer behavior (see the later discussion).

As it is commonly believed that increasing the charge state leads to an increase in CCS due to Coulombic repulsion, our data also show that the gas-phase [1]^2+^ and [1]^3+^ species exhibit markedly different behavior. Specifically, the higher-charged peptide ion ([1]^3+^) adopts a more extended conformation, resulting in a larger CCS, consistent with enhanced Coulombic repulsion. Simulation trajectories and structural comparisons for both charge states are provided in SI section S4. For other oligomeric charge states, our analysis primarily focuses on the most abundant species, as these are likely to represent dominant conformations evolved from the solution state. Nonetheless, we acknowledge that minor conformations, whether arising from less prevalent charge states or from structural heterogeneity within a single charge state, may also contribute to the oligomerization process. However, quantitatively tracking these low-abundance species is challenging owing to their intrinsic scarcity. These minor species often appear as small dots above the main trendline with elevated CCS values, suggesting looser conformations that may represent intermediates or minor byproducts from the oligomerization processes.

Overall, simulated CCS values, indicated by “×” markers along the trendline, show good agreement with experimental measurements, with most values falling within a 15% deviation based on the IMPACT TJM method. However, it failed to produce consistent CCS values for low mass ions. The dimer shows a discrepancy of up to 38%, and the monomer exhibits a deviation reaching as high as 50%. This deviation is unlikely to originate from the calculation algorithm itself; instead, it is more likely due to an underestimated experimental value or an overextended input structure.

We also benchmarked several CCS calculation methods to determine which one provides the best agreement with the experimental data. The overall trend in absolute percentage deviation is as follows: PSA > IMPACT PA > PA ≈ IMPACT TJM, for oligomers with *n* > 4. However, for smaller oligomers (*n* = 1–3), all four methods yield significantly higher CCS values compared to the experiment, except for IMPACT PA, which provides relatively closer estimates (see SI section S5). To maintain consistency across data analysis, we chose to use the IMPACT TJM method, as it offers better alignment with the experimental results in most cases.

The large deviation observed for small oligomers may be attributed to two main factors. First, the monomer and dimer conformations observed in the simulations exhibited a range of structural variations. As a result, the predicted CCS values for smaller oligomers could deviate without accounting for their microstate distribution. Second, the calibration profile for the G2 instrument may lack a sufficient dynamic range to accurately cover smaller oligomers, which requires the need for improved calibrants for small peptides.

A significant amount of research has focused on reproducing CCS profiles and aligning predicted CCS values with experimental results.^56–58^ Here, however, we concentrated on identifying general trends rather than achieving precise numerical matches for CCS values. Based on the CCS values obtained, linear, isotropic, and anisotropic growth models were applied to fit the CCS profiles of oligomers (Figure 5A). The fitted parameter, **σ**, reflects the extent of deviation from a spherical structure, where higher **σ** values indicate a more anisotropic growth, i.e., the formation of more ellipsoidal shapes.

Our analysis of the fitted **σ** values reveals distinct growth patterns across different oligomer sizes. For oligomers ranging from 2-mer to 16-mer, the fitted **σ** value is 1.28. However, some minor conformations, such as those from the 2-mer, 4mer, and 5-mer, fall below the fitted curve, while others, including the 7-mer to 9-mer and 11-mer to 16-mer, lie above it. This suggests that although these oligomers grow anisotropically at a specific mode, the degree of anisotropy varies among them. Conformations above the fitted curve indicate more ellipsoidal shapes, whereas those below suggest more spherical geometries. Notably, the larger oligomers (*n* = 27, 28, and 29) observed by nIM-MS exhibit significantly higher **σ** values and have more minor distributions of extended conformations, implying that they may assemble via a fundamentally distinct mechanism that produces markedly different shapes compared to smaller oligomers.

Another important consideration is the comparison between realistic and theoretical models. As mentioned earlier, the fitting model assumes that the object is a convex body. If this assumption is not met, the fitted **σ** value, whether derived from experimental or simulated CCS data, will deviate from the theoretical **σ**.

By leveraging the PSA method, we can quantitatively assess surface ruggedness in terms of convexity. This metric reflects how much a surface deviates from an ideal convex shape. Specifically, convexity is calculated as the ratio of the solvent-excluded surface area to the surface area of the smallest enclosing convex hull. PSA calculations reveal that the shape factor increases from 1.11 for the monomer to 1.30 for the 7-mer, indicating a trend toward rougher surfaces as the oligomer size increases. Larger oligomers are surprisingly smoother than the 7-mer, as indicated by their shape factors, with values of 1.20 for the 14-mer and 1.22 for the 30-mer (SI Table S2). This suggests a convergence toward a consistent degree of surface roughness. These results imply that small oligomers may deviate from the ideal convex shape to varying extents, whereas larger oligomers exhibit a more uniform deviation from this assumption. Thus, although small oligomers show high **σ** values from the fitting, this does not necessarily imply a strongly ellipsoidal shape. In fact, they may be closer to spherical shapes, as their deviation from the ideal convex assumption could inflate the fitted **σ**. In contrast, larger oligomers such as the 14-mer and 30-mer, which display relatively smoother surfaces, are more reliably associated with a highly anisotropic growth mode.

### Monomer Conformation in Free and Oligomeric States

Returning to our central question about the factors influencing oligomerization raises further questions about the role of conformation: is there a preferred monomer conformation that leads to oligomerization? In other words, what is the conformational difference between free monomers and the individual monomers within oligomers? To address this problem, we used the radius of gyration (Rg) as an indicator of monomer conformation, allowing us to compare the structural characteristics of monomers within oligomers with those of free monomers.

For a free monomer, enhanced sampling via REMD was employed to explore a wide range of conformational states, resulting in four distinct clusters categorized by their structural features (Figure 6A). These clusters are referred to as compact, extended, twisted, and unfolded conformational states (Figure 6B), collectively providing a comprehensive view of the monomer’s conformational landscape.

**Figure 6:**
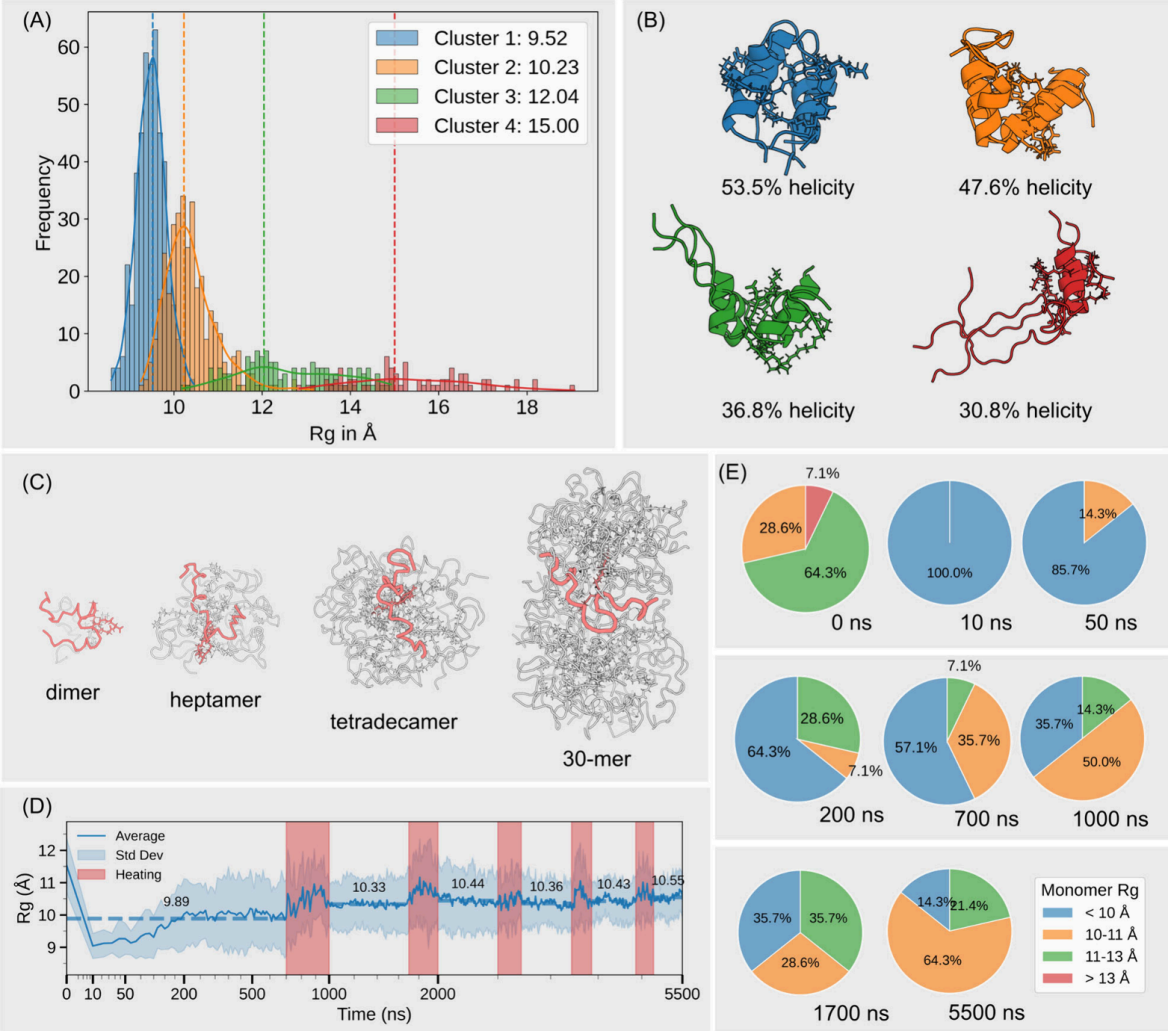
(A) Clustering of free monomer conformations with centroid Rg values shown in the legend. (B) Representative conformations from the clustering, with the fatty tail highlighted as sticks. (C) Graphic representation of monomers highlighted within different oligomers, representing conformational differences of monomers. The monomer expands as the oligomer grows. (D) Averaged monomer Rg over time for a tetradecamer; the time axis is warped for clarity, and heating regions are highlighted in red. (E) Time-stamped tracking of specific monomer conformation percentage.

The compact cluster, characterized by a low Rg value, primarily consists of loop-helix-loop conformations. In this state, the peptide adopts a tightly folded structure in which two helical regions interact closely, stabilized by intramolecular hydrophobic interactions. This configuration resembles a “hand grip” and represents the most energetically favorable state. It is highly populated (45.57% abundance) and exhibits the highest helicity percentage (53.5%) among all clusters. Moving to the middle range of Rg, the second most populated region (34.51% abundance) corresponds to the extended cluster, which is characterized by increased spatial expansion and reduced helicity. These bent structures represent transition conformations that are structurally between the compact and twisted states. At the right end of the Rg dimension lies the twisted cluster (12.37% abundance), characterized by partially unfolded conformations with a significant helicity loss. Beyond this, we have also sampled a small population of disordered conformation at the rightmost end of the Rg dimension, comprising 7.55% of the total population. These high-energy states are sparsely populated due to their thermodynamic instability, resulting from the loss of stabilizing intramolecular interactions such as hydrogen bonding and hydrophobic core packing.

To investigate how individual monomers behave within oligomers (illustrative examples are shown in Figure 6C), we examined the monomer conformations across different time points. Taking the Path I product, a compact 14-mer as an example, we shift focus from the global Rg of the entire oligomer (as shown in Figure 2B) to the average monomer Rg, calculated on a per-monomer basis. Specifically, at each time point, individual monomers were extracted from the whole oligomer; their Rg values were computed separately, and the average was taken across all monomers. This analysis, shown in Figure 6D, provides insight into the average monomer size as well as the conformational heterogeneity among monomers, as indicated by the standard deviation (represented by the blue shaded band).

Unlike Figure 2B, which displays a continuous decline in the overall oligomer Rg, Figure 6D reveals a more complex pattern. The average monomer Rg drops sharply within the first 10 ns, followed by a substantial increase over the next 700 ns. Beyond this point, during the subsequent five annealing cycles, the average Rg fluctuates but shows a slight, gradual upward trend. Concurrently, the standard deviation band also narrows progressively.

These observations support the following conclusions: (1) Early compaction: at early time points (e.g., 10 ns), monomers tend to adopt compact conformations, particularly when in a free state, as evidenced by the percentage reported in Figure 6E. (2) Conformational shift during oligomerization: as oligomerization proceeds, a greater proportion of monomers adopt “extended” or “twisted” conformations, as shown in the pie charts at 50, 200, 700, and 1000 ns. (3) Final state preference for monomer conformation: by the final stage, the system stabilizes, with 64.3% of monomers occupying extended conformational states. (4) Reduced conformational heterogeneity: the narrowing of the standard deviation band suggests reduced conformational heterogeneity, indicating that extremely disordered or overly compact monomers become less prevalent over time, with the population increasingly centered around the extended conformation state.

To avoid potential misunderstandings, it is important to clarify the basis of conformation classification. For the free monomer, the K-means algorithm was applied and ultimately identified four structural clusters with various ranges of Rg values (see SI section S7 for method details). However, this clustering approach cannot be directly applied to individual monomers within oligomers. Specifically, not all monomers from the 14-mer can be reliably assigned to the predefined clusters. For example, the structural features of a given peptide may not precisely match the criteria established from the free monomer data set. While the free monomer classification was based on 1000 conformations, the oligomeric monomer data set has a limited sample size (only 14 monomers analyzed at each time point), which prevents reliable and consistent clustering. As a result, we adopted a fixed boundary definition to classify monomer conformations within oligomers, which represents a practical compromise yet still provides a sufficient basis for capturing the main conformational trends.

So far, our analysis shows that free monomers exhibit diverse conformational populations. To evolve into large oligomers, these monomers must adopt more extended conformations. Notably, the average Rg of monomers within the final equilibrated 14-mer closely matches the Rg of the extended conformational population observed in the free monomer ensemble. This similarity suggests that prepopulating extended states may promote oligomer formation. Since extended peptides can assemble without requiring significant structural rearrangements, the assembly process is likely more energetically favorable when extended states are more populated.

## Conclusion

In this work, we investigated the phenomenon of liraglutide oligomerization through simulations and validated our findings with experimental results. We found that the hydrophobic effect, primarily driven by the fatty tail, dominates the liraglutide oligomerization process. Additionally, our proposed anisotropic growth model provides a foundation for further CCS interpretation in the field of ion mobility mass spectrometry. The distortion factor **σ**, derived from the CCS trendline fitting, offers structural insights beyond what a single CCS value can provide. Our data indicate that liraglutide oligomerizes anisotropically to varying degrees at different oligomerization stages. Larger oligomers (*n* = 26–29) adopt more ellipsoidal shapes, while smaller oligomers (*n* < 15) are more spherical and exhibit greater structural dynamics. A closer examination of simulated monomer conformations reveals that monomer conformation is directly related to oligomer size, with extended conformations being more prevalent in larger oligomers.

Another important insight from the Rg analysis is its implication for a potential upper limit of oligomer size. As shown in the SI (Table S3), the average monomer Rg converges around 10.5 Å even as the oligomers increase in size. More interestingly, the size distribution of the detected oligomers is not continuous. For instance, oligomers between 17-mer and 26-mer were not observed by nIM-MS. This discontinuity may indicate the presence of a critical oligomer size, possibly representing a structural limit where a single hydrophobic core can accommodate the maximum number of subunits. Upon reaching this threshold, liraglutide may shift to an alternative oligomerization mode, resulting in the formation of larger assemblies that emerge in discrete steps rather than a continuous progression, likely adopting different structural characteristics.

Future studies will explore how modified residues influence peptide conformations and, consequently, the propensity for oligomerization. Additional research will also investigate the effects of environmental factors, such as solvent polarity, osmolyte levels, temperature, and pH, which may provide further insights into the oligomerization process.

## Supplementary Material



## Notes

Details on mass spectra, CCS data, simulation setup, additional 14-mer trajectories, CCS benchmarking, monomer charge state comparison, conformation clustering, monomer Rg monitoring, the anisotropic growth model, the fuzzy oil drop model, and the free energy landscape (PDF)
