# Supplementary material for: Revealing Anisotropic Growth of Liraglutide Oligomers by Native Ion Mobility Mass Spectrometry and Molecular Dynamics Simulation
Source: ACS Cent Sci. 2025 Jun 16;11(7):1154–65. doi: 10.1021/acscentsci.5c00431 (PMC12291117; doi:10.1021/acscentsci.5c00431)
Supplement: Supplementary file 1 [file oc5c00431_si_001.pdf]

Supporting Information

Revealing Anisotropic Growth of Liraglutide Oligomers by Native Ion Mobility Mass Spectrometry and Molecular Dynamics Simulation

Zhenyu Xi[1], Syuan-Ting Kuo[1], Xiao Cong[2], Xin Yan[1], and David H. Russell[1*]

[1]Department of Chemistry, Texas A&M University, College Station, Texas 77843, USA

[2]Boehringer Ingelheim, Ridgefield, Connecticut, 06877, USA

*Corresponding author; email: russell@chem.tamu.edu

Contents

S1. Mass spectra and CCS data S2
Figure S1. .... S2
Table S1 .... S3
Figure S2. .... S7
Figure S3. .... S8
S2. Simulation Setup S9
Figure S4. .... S9
S3. Additional two simulation trajectories of 14-mer formation S11
Figure S5. .... S11
Figure S6. .... S12
S4. Monomer at 2+ and 3+ charge state S13
Figure S7. .... S13
S5. CCS Benchmarking S14
Table S2 .... S14
Figure S8. .... S19
Figure S9. .... S19
S6. Conformation clustering S20
S7. Monitoring of monomer Rg S21
Table S3 .... S21
Appendix I. Anisotropic Growth Model S22
Table S4 .... S25
Appendix II: Fuzzy Oil Drop Model S26
Appendix III. Free Energy Landscape S30
Reference S36

S1. Mass spectra and CCS data

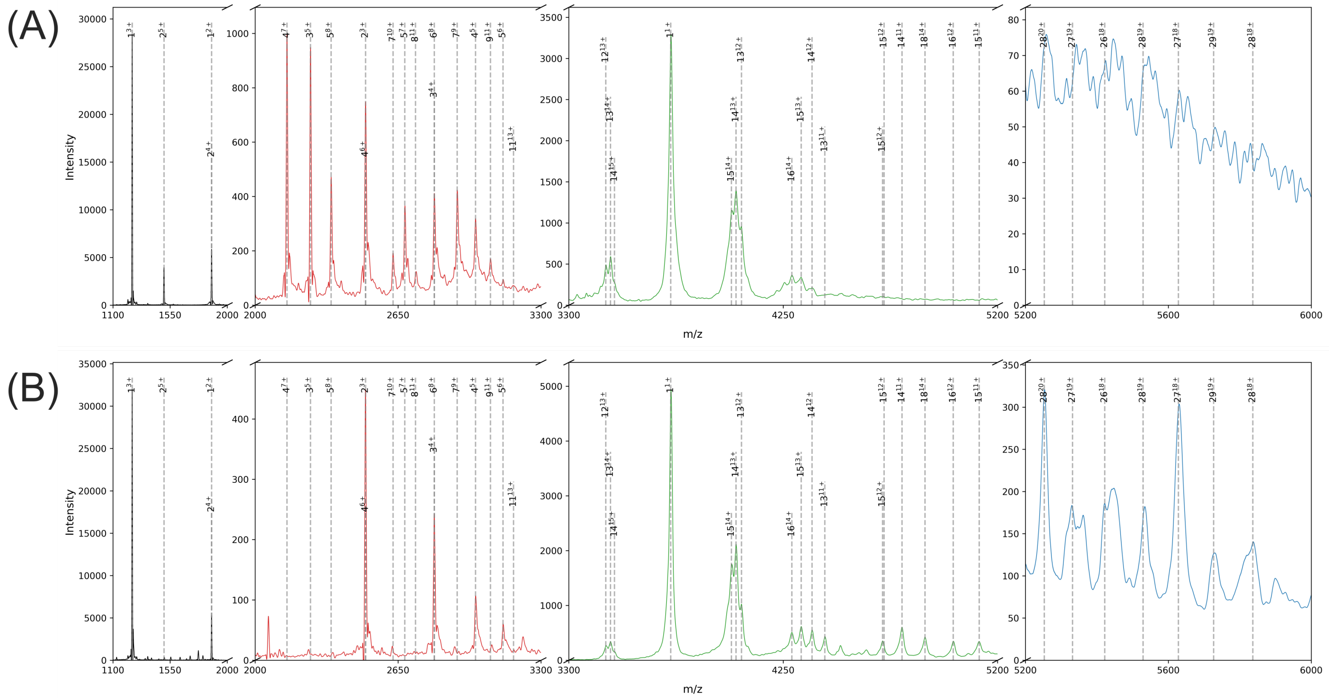

Figure S1. Mass spectra showing the effect of transfer collision energy on oligomer transmission. (A) At 0 V, smaller oligomers (black and red regions) are better transmitted. (B) At 50 V, larger oligomers (green and blue regions) are better transmitted.

Table S1. CCS peak values of oligomers.

Entry	Peak Index	m/z	Relative Intensity	Oligomeric State	Charge	CCS
1	1	1876.64	100.00	1	2	439.67
2	1	1251.42	100.00	1	3	558.75
3	2	1251.42	94.58	1	3	627.26
4	1	2501.85	100.00	2	3	710.98
5	1	1876.64	46.89	2	4	788.76
6	1	1501.51	100.00	2	5	928.95
7	2	1501.51	95.26	2	5	1042.96
8	1	2251.76	2.22	3	5	1003.57
9	2	2251.76	100.00	3	5	1184.62
10	3	2251.76	3.25	3	5	1450.74
11	4	2251.76	1.29	3	5	1560.25
12	1	2501.85	29.60	4	6	1248.21
13	1	2144.58	100.00	4	7	1431.06
14	1	3127.06	100.00	5	6	1398.28
15	2	3127.06	8.86	5	6	1661.47
16	3	3127.06	5.59	5	6	1776.61
17	1	2680.48	100.00	5	7	1632.21
18	2	2680.48	1.13	5	7	2095.5
19	1	2345.54	100.00	5	8	1664.38
20	2	2345.54	33.90	5	8	1894.21
21	1	2918.65	100.00	7	9	2097.49
22	1	2626.89	100.00	7	10	2115.99
23	2	2626.89	66.39	7	10	2296.14
24	3	2626.89	14.96	7	10	2538.05
25	4	2626.89	1.78	7	10	2704.55
26	1	2729.19	100.00	8	11	2602.54
27	2	2729.19	21.43	8	11	2828.41
28	1	3070.22	100.00	9	11	2485
29	2	3070.22	3.93	9	11	2973.35
30	3	3070.22	2.85	9	11	3325.15
31	1	3175.15	0.82	11	13	2603.15
32	2	3175.15	100.00	11	13	2842.89
33	3	3175.15	6.78	11	13	3470.55
34	1	3463.71	100.00	12	13	3027.13

35	2	3463.71	5.65	12	13	3639.31
36	1	4434.31	3.45	13	11	2896.8
37	2	4434.31	100.00	13	11	3182.76
38	3	4434.31	7.41	13	11	3624.11
39	4	4434.31	11.29	13	11	3787.36
40	5	4434.31	11.04	13	11	3947.45
41	6	4434.31	2.95	13	11	4259.11
42	7	4434.31	3.45	13	11	4380.87
43	8	4434.31	1.01	13	11	4590.33
44	1	4064.87	100.00	13	12	3161.13
45	2	4064.87	3.02	13	12	3918.4
46	3	4064.87	0.71	13	12	4546.36
47	1	3484.32	100.00	13	14	3259.83
48	2	3484.32	2.33	13	14	4009.07
49	1	4377.48	100.00	14	12	3317.88
50	2	4377.48	7.38	14	12	3734.95
51	3	4377.48	5.83	14	12	3989.53
52	4	4377.48	2.20	14	12	4340.89
53	5	4377.48	0.93	14	12	4646.33
54	1	4040.82	100.00	14	13	3337.7
55	1	3502.18	100.00	14	15	3545.06
56	2	3502.18	2.47	14	15	4438.06
57	1	4690.08	5.32	15	12	3079.18
58	2	4690.08	5.90	15	12	3238.64
59	3	4690.08	100.00	15	12	3509.43
60	4	4690.08	29.05	15	12	3697.12
61	5	4690.08	8.80	15	12	4166.12
62	6	4690.08	13.43	15	12	4340.12
63	7	4690.08	11.46	15	12	4876.93
64	8	4690.08	4.28	15	12	5262.35
65	1	4329.38	100.00	15	13	3466.71
66	2	4329.38	0.63	15	13	4887.76
67	1	4020.21	100.00	15	14	3452.03
68	1	4288.16	100.00	16	14	3640.46
69	2	4288.16	10.83	16	14	4095.43
70	3	4288.16	2.76	16	14	4654.54
71	1	5627.9	100.00	27	18	5486.13

72	2	5627.9	30.63	27	18	6033.04
73	3	5627.9	24.29	27	18	6402.7
74	4	5627.9	6.83	27	18	6762.85
75	5	5627.9	5.40	27	18	7409.59
76	6	5627.9	1.27	27	18	7937.27
77	1	5331.74	100.00	27	19	5553.62
78	2	5331.74	95.74	27	19	5967.78
79	3	5331.74	14.35	27	19	6704.26
80	4	5331.74	19.28	27	19	6868.94
81	5	5331.74	5.61	27	19	7139.49
82	6	5331.74	3.81	27	19	7718.92
83	7	5331.74	6.50	27	19	11387.15
84	1	5836.3	100.00	28	18	5596.76
85	2	5836.3	66.89	28	18	6085.8
86	3	5836.3	12.61	28	18	6711.3
87	4	5836.3	3.38	28	18	7408.97
88	5	5836.3	4.73	28	18	7746.68
89	1	5529.18	100.00	28	19	5672.63
90	2	5529.18	41.38	28	19	6140.58
91	3	5529.18	24.01	28	19	6368.5
92	4	5529.18	8.94	28	19	6922.77
93	5	5529.18	7.15	28	19	7298.94
94	6	5529.18	3.96	28	19	7821.49
95	7	5529.18	6.77	28	19	9111.88
96	8	5529.18	0.64	28	19	9350.36
97	1	5252.77	100.00	28	20	5718.9
98	2	5252.77	25.00	28	20	6159.02
99	3	5252.77	33.04	28	20	6645.05
100	4	5252.77	20.54	28	20	6940.7
101	5	5252.77	3.13	28	20	7230.7
102	6	5252.77	16.96	28	20	7515.48
103	1	5726.61	100.00	29	19	6024.47
104	2	5726.61	21.50	29	19	6647.61
105	3	5726.61	14.69	29	19	6922.12
106	4	5726.61	8.22	29	19	7351.31
107	5	5726.61	1.92	29	19	7717.65
108	6	5726.61	8.04	29	19	7923.46

109	7	5726.61	4.55	29	19	8723.63
110	8	5726.61	1.57	29	19	9015.09

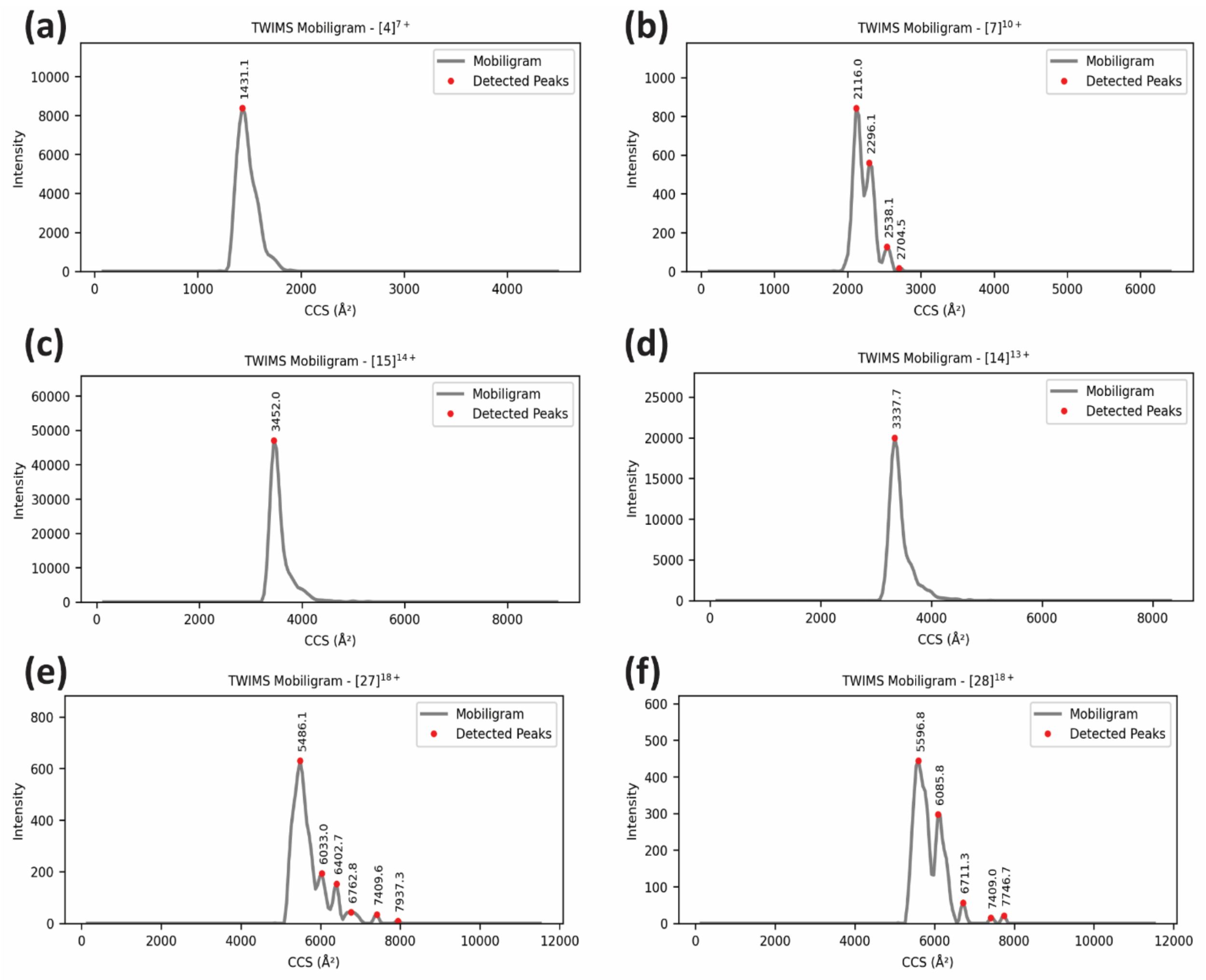

Figure S2. Representative extracted ion mobilogram (EIM) of liraglutide oligomers. (a) $[4]^{7+}$ (m/z 2144) (b) $[7]^{10+}$ (m/z 2627) (c) $[15]^{14+}$ (m/z 4020) (d) $[14]^{13+}$ (m/z 4041) (e) $[27]^{18+}$ (m/z 5628) (f) $[28]^{18+}$ (m/z 5836). Quadrupole isolation was implemented for each *m/z* prior to mobility analysis to prevent the detection of products from other oligomers (see Figure S4). The mobilograms were smoothed using a Savitzky-Golay filter in MassLynx.

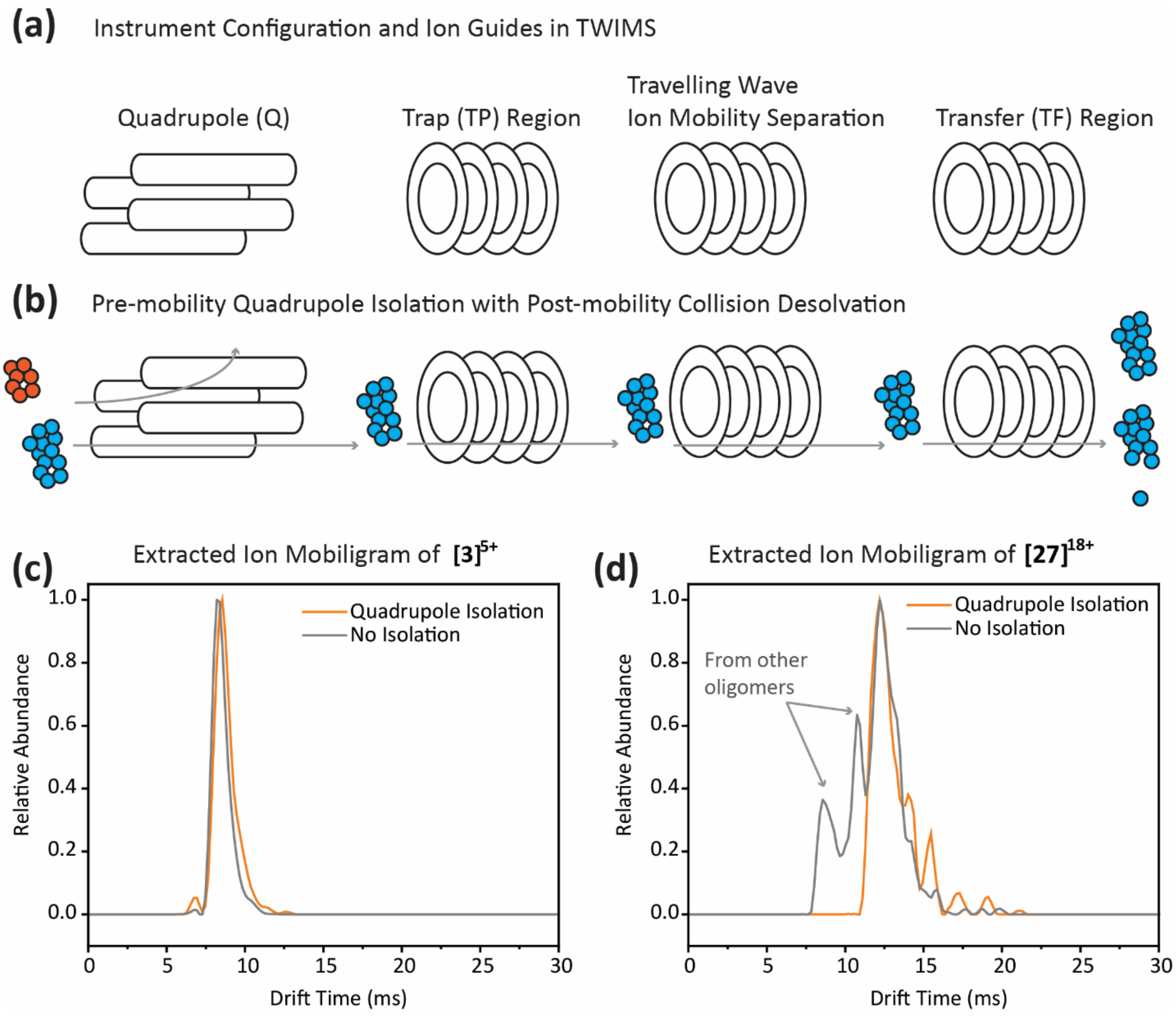

Figure S3. Pre-mobility Isolation for Collision Cross Section (CCS) Determination. (a) Instrument configuration of the Waters Synapt G2 Mass Spectrometer. (b) Schematic of pre-mobility isolation by quadrupole. Quadrupole isolation allows the entry of a specified m/z into the mobility cell, reducing the likelihood that the measured m/z originates from other precursor ions. (c) Extracted ion mobilogram of $[3]^{5+}$ under no transfer energy, with and without quadrupole isolation. (d) Extracted ion mobilogram of $[27]^{18+}$ under 50 V transfer energy, with and without quadrupole isolation.

S2. Simulation Setup

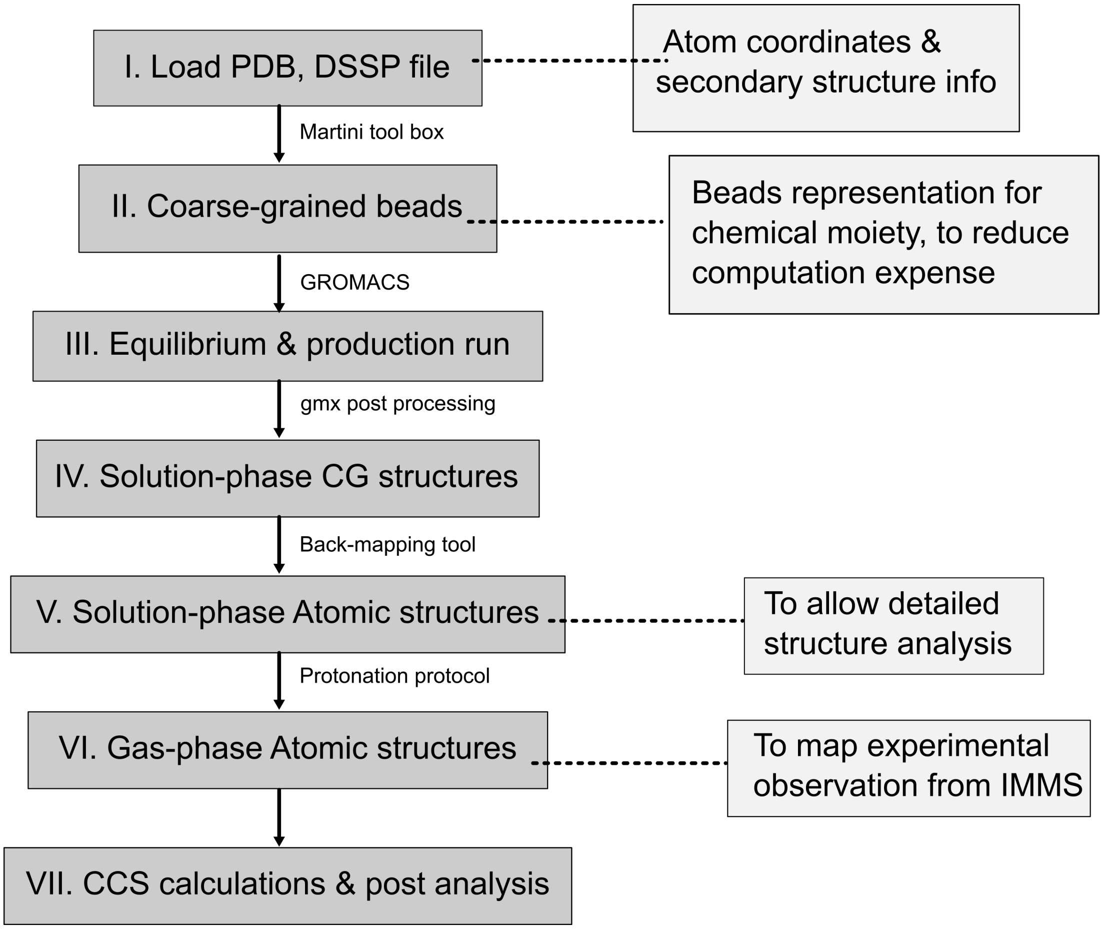

Figure S4. Simulation workflow

Coarse-grained (CG) simulations were performed using the Martini 2.2 force field[1, 2], and atomistic simulations employed the CHARMM36m force field[3], both implemented in GROMACS 2023.3[4]. As required by CG simulations, peptide secondary structure information was specified. The input secondary structure was identified using the DSSP package[5], based on the first frame of the solution NMR structure 4apd.pdb. The nonstandard residue, D6M, was parameterized using CHARMM-GUI[6] and subsequently converted to CG resolution using the CGbuilder tool[7]. To back-map from CG resolution to atomic resolution, a backward mapping script was used.[8]

Simulations were conducted for 2-mer, 3-mer, 4-mer, 5-mer, 6-mer, 7-mer, and 14-mer peptides, each performed in triplicate. Each system consisted of the corresponding number of liraglutide molecules randomly placed in a periodic cubic box, ensuring a minimum separation of 2 nm between peptides. Simulations began with energy minimization to resolve any unfavorable contacts, followed by 1 ns of NVT equilibration and 1 ns of NPT

equilibration. Production simulations employed simulated annealing, consisting of five annealing cycles. Each annealing cycle consisted of 700 ns at 300 K followed by 300 ns at 360 K. After five such cycles, an additional 500-ns simulation was performed at 300 K, resulting in a total simulation time of 5500 ns. For the 30-mer peptides, simulations were performed in duplicate using the same protocol as for smaller oligomers but extended to nine annealing cycles to ensure structural equilibration, yielding a total of 9500 ns per replicate.

The gas-phase simulation was performed in a 999 × 999 × 999 nm^3 periodic cubic box, with all cut-offs set to 333.3 nm. Translational and rotational velocities around the protein's center of mass were removed every 100 steps to stabilize the system.

Temperature coupling was controlled using the v-rescale thermostat, while pressure was maintained at 1 bar using the Berendsen barostat for equilibration and the Parrinello-Rahman barostat for production simulations.[9, 10] The CG simulation time step was set to 25 fs, while the atomic level simulation time step was set to 2 fs. Water beads were mixed with 10% anti-freeze beads as the solvent, and chloride ions were added to neutralize the system. The solution concentration for simulated peptides was maintained at 3 mM as a compromise between minimizing computational cost and approximating the conditions of experimental oligomerization solutions.

A replica exchange molecular dynamics (REMD) simulation was conducted to comprehensively sample monomer conformations. The temperature ladder was designed to achieve an exchange probability of 0.3[11], with the following temperatures: 300.00, 302.39, 304.79, 307.22, 309.62, 312.08, 314.55, 317.04, 319.54, 322.06, 324.60, 327.15, 329.73, 332.32, 334.92, 337.54, 340.18, 342.84, 345.51, 348.21, 350.92, 353.65, 356.40, 359.16, and 360.00 K. The simulation box dimensions were set to 7.22 × 3.85 × 3.94 nm^3. The production run was performed for 1,000 ns.

S3. Additional two simulation trajectories of 14-mer formation

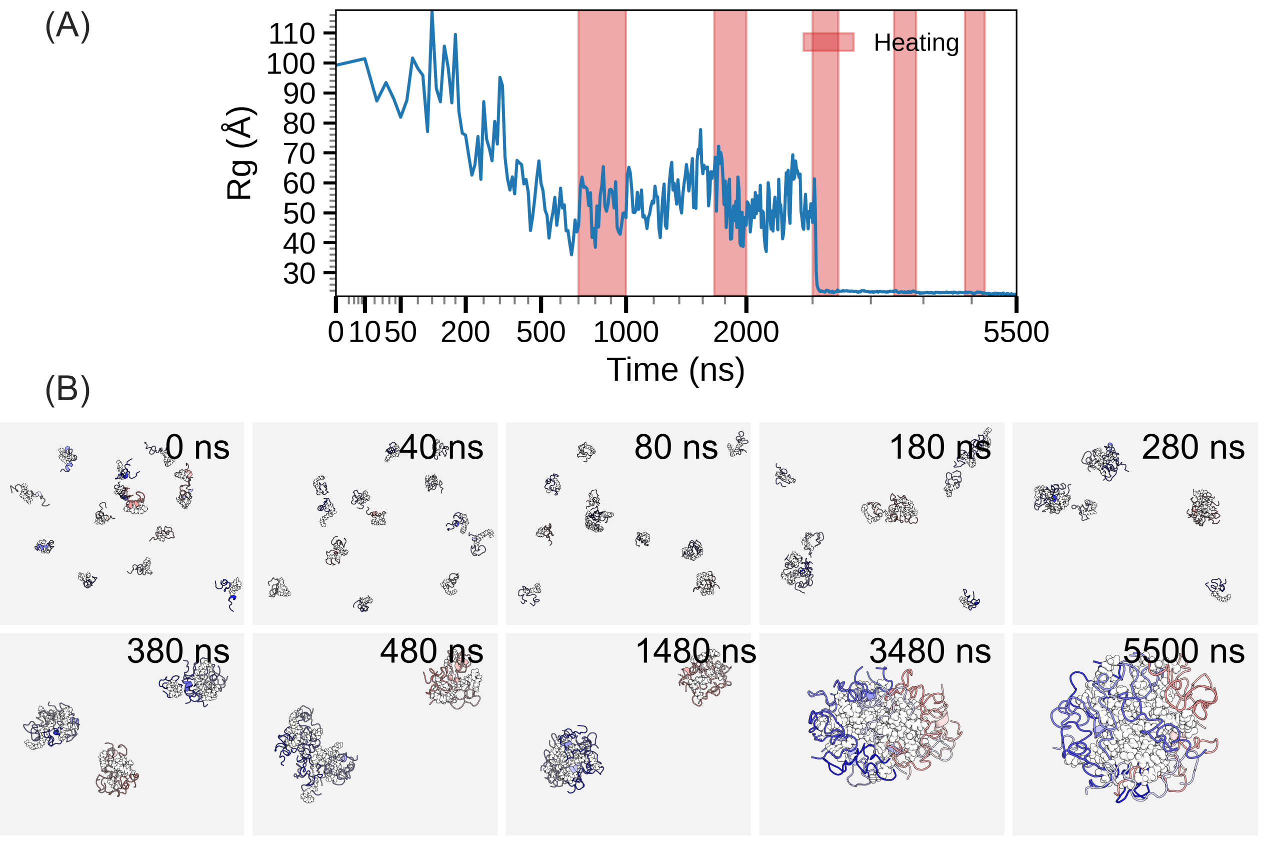

Figure S5. Monitoring the assembly process of liraglutide over time in replicate 2. (A) Time evolution of the overall radius of gyration, with periodic simulated annealing applied. (B) Representative structures of the 14-liraglutide assembly at selected time points during the simulation.

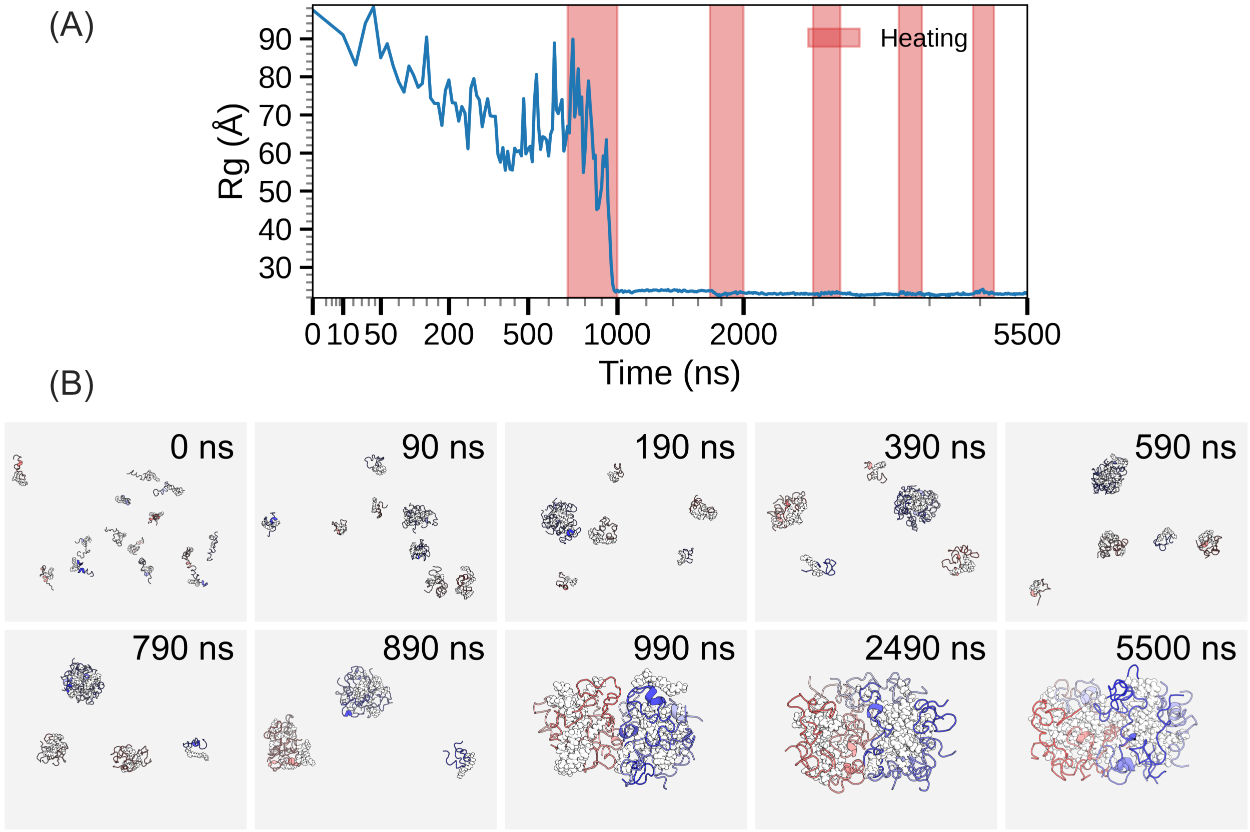

Figure S6. Monitoring the assembly process of liraglutide over time in replicate 3. (A) Time evolution of the overall radius of gyration, with periodic simulated annealing applied. (B) Representative structures of the 14-liraglutide assembly at selected time points during the simulation.

S4. Monomer at 2+ and 3+ charge state

Gas-phase simulations for the 2 charge states were initiated from appropriately protonated, solution-phase structures, following the simulation protocol described in Section S2 and the protonation protocol detailed in the main text. The starting structure, obtained from the solution NMR structure 4APD.pdb, is shown in blue in the aligned trajectory snapshots. Structures become progressively redder to indicate later simulation times. Each simulation was run for 10 ns, during which no further structural change was observed in the last 5 ns. While these two simulations reflect the gas-phase evolution of a single solution conformation, a more rigorous approach would require hundreds or thousands of simulations starting from a diverse ensemble of solution structures that correctly follow the microstate distribution of the solution-phase conformational ensemble. Although only a single run was carried out for each of the two charge states, gas-phase compaction was observed for the 2+ ion, while the 3+ ion remained extended due to Coulombic repulsion.

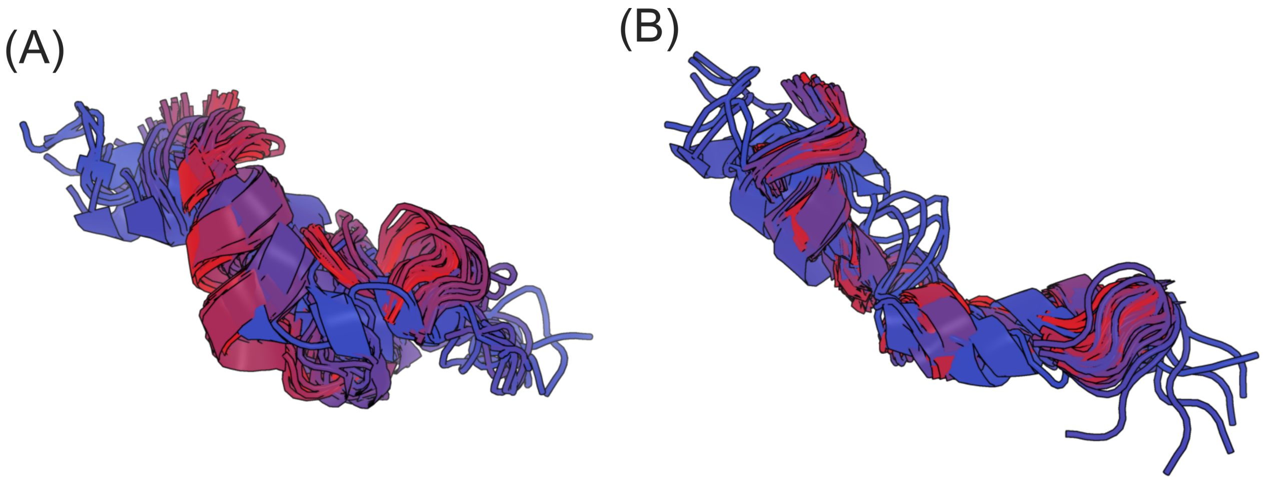

Figure S7. Structural evolution of $[1]^{2+}$ and $[1]^{3+}$ species during transfer from solution to gas phase. Conformations are aligned and colored from blue to red, representing the progression from the initial to final state. (A) 2+ charge state. (B) 3+ charge state.

S5. CCS Benchmarking

Table S2. Oligomer structures used as input for CCS calculations, with corresponding CCS values and shape factor values.

Dimer	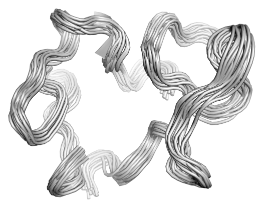	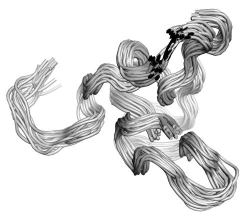	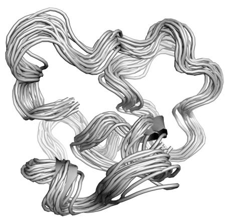
$[2]^{3+}$ Experimental CCS ($Å^2$)	710.98		
IMPACT_PA	833.80±6.97	824.04±8.23	815.02±8.92
IMPACT_TJM	991.93±8.72	979.73±10.29	968.46±11.14
PA	1069.52±12.16	1053.57±16.93	1053.33±20.36
PSA	1218.4±17.1	1203.6±21.7	1210.0±26.3
Shape factor	1.139±0.007	1.142±0.006	1.149±0.008
Trimer	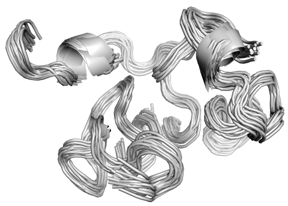	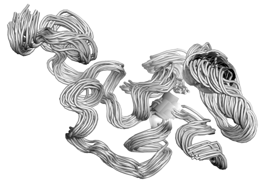	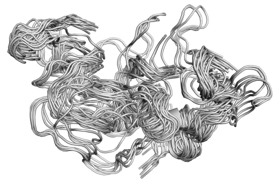
$[3]^{5+}$ Experimental CCS ($Å^2$)	1184.62		
IMPACT_PA	1050.42±7.77	1120.09±12.34	1179.70±36.45

IMPACT_TJM	1264.53±9.83	1352.85±15.67	1428.67±46.39
PA	1327.92±15.97	1427.14±28.52	1487.20±56.29
PSA	1555.0±17.1	1626.6±30.7	1711.3±58.9
Shape factor	1.171±0.007	1.140±0.009	1.151±0.011
Tetramer	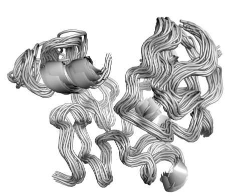	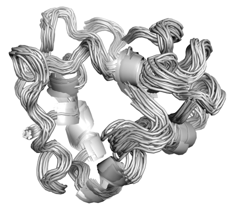	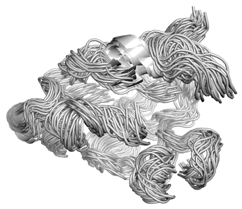
$[4]^{7+}$ Experimental CCS (Å^2)	1431.06		
IMPACT_PA	1233.87±12.60	1202.09±6.45	1221.96±10.92
IMPACT_TJM	1497.68±16.08	1457.16±8.22	1482.48±13.92
PA	1537.40±24.09	1505.22±18.59	1523.07±25.57
PSA	1775.9±34.2	1757.5±22.0	1744.7±32.9
Shape factor	1.155±0.013	1.168±0.012	1.146±0.017
Pentamer	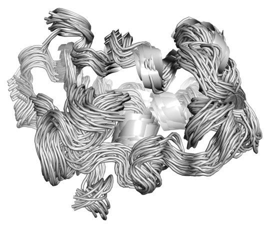	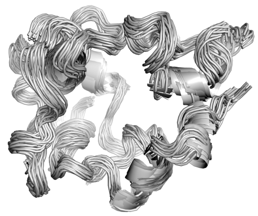	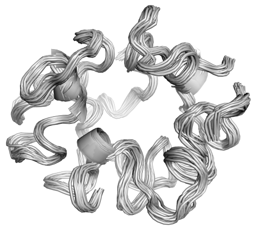

$[5]^{7+}$ Experimental CCS (Å^2)	1632.21		
IMPACT_PA	1465.21±11.29	1468.05±10.24	1368.54±7.01
IMPACT_TJM	1794.22±14.54	1797.87±13.18	1669.99±8.99
PA	1815.13±32.12	1826.01±26.44	1690.09±20.94
PSA	2206.9±103.7	2199.8±72.3	2048.2±61.8
Shape factor	1.216±0.047	1.205±0.037	1.212±0.037
Hexamer	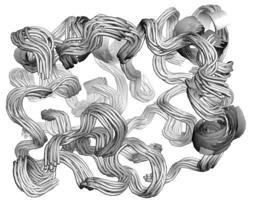	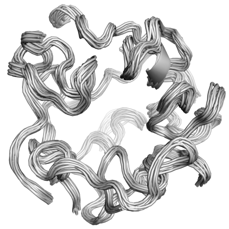	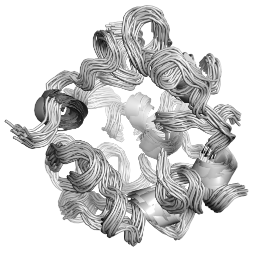
$[6]^{8+}$ Experimental CCS (Å^2)	1864.842		
IMPACT_PA	1559.35±8.89	1528.42±8.33	1548.17±7.91
IMPACT_TJM	1915.61±11.49	1875.69±10.75	1901.17±10.22
PA	1893.86±24.75	1857.04±9.75	1887.01±11.38
PSA	2419.3±165.4	2342.3±141.6	2345.2±124.9
Shape factor	1.277±0.085	1.261±0.074	1.243±0.066

Heptamer	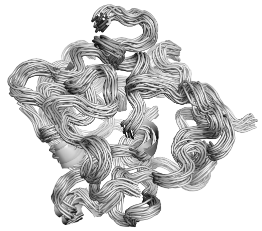	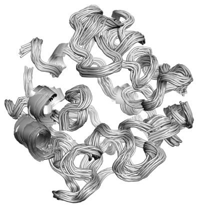	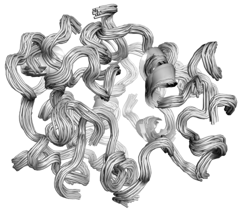
$[7]^{9+}$ Experimental CCS (Å^2)	2097.49		
IMPACT_PA	1720.62±8.56	1705.11±8.99	1720.86±9.33
IMPACT_TJM	2124.42±11.11	2104.29±11.66	2124.73±12.12
PA	2087.09±20.59	2085.10±26.99	2070.17±30.57
PSA	2735.3±336.9	2676.8±601.6	2712.1±345.7
Shape factor	1.311±0.163	1.284±0.288	1.310±0.163
	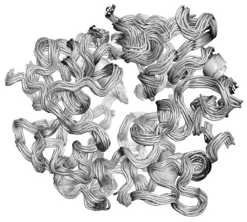	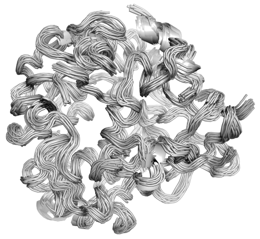	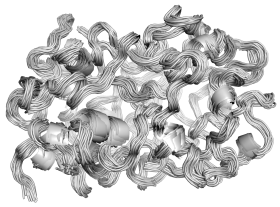
$[14]^{13+}$ Experimental CCS (Å^2)	3337.705		
IMPACT_PA	2609.89±12.22	2632.68±11.66	2691.78±13.10
IMPACT_TJM	3291.97±16.20	3322.19±15.46	3400.64±17.40
PA	3060.38±42.03	3105.22±30.29	3150.71±40.29

PSA	3664.2±49.3	3771.4±39.0	3750.5±63.1
Shape factor	1.197±0.006	1.215±0.005	1.190±0.008
$[30]^{20+}$ *	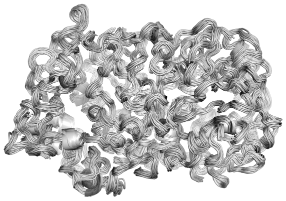	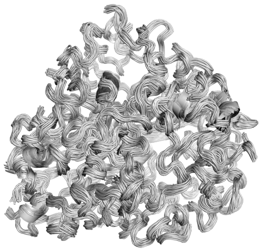	
IMPACT_PA	4488.41±19.17	4354.65±16.86	
IMPACT_TJM	5821.04±26.13	5638.81±22.95	
PA	5114.17±65.19	4972.48±63.12	
PSA	6196.3±77.4	6143.8±83.6	
Shape factor	1.212±0.004	1.236±0.013	

*30-mer is not observed by IMMS

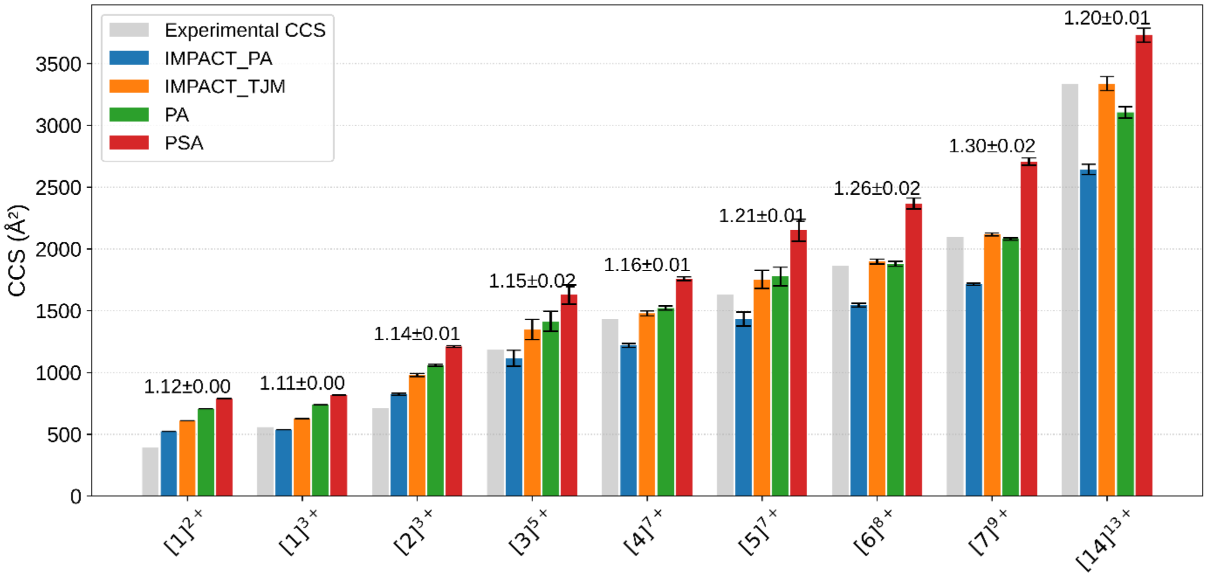

Figure S8. CCS value comparison among experimental values and calculated values. Shape factors are denoted at the top of each group.

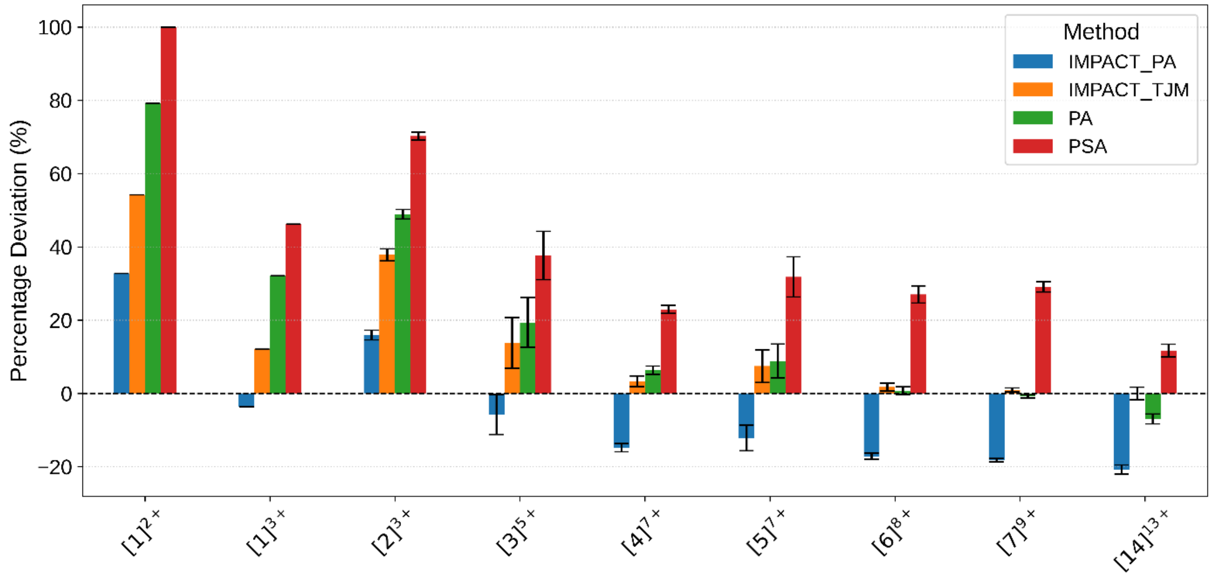

Figure S9. Percentage deviation calculated from different calculation methods.

S6. Conformation clustering

Principal component analysis (PCA) was used to visualize the oligomerization pathway and identify key residues influencing the process. The absolute coordinates of Cα atoms were extracted and normalized as input features. Residues' contributions to oligomerization were calculated by normalizing the absolute values of their covariances. The K-means algorithm was employed to identify representative structures based on a set of features, including the radius of gyration (Rg) of all peptides, Rg of hydrophobic residues, Rg of hydrophilic residues, solvent-accessible surface area (SASA) of all peptides, SASA of hydrophobic residues, SASA of hydrophilic residues, end-to-end distance (Ree) for each chain, and helicity percentage from DSSP.[12]

S7. Monitoring of monomer Rg

Table S3. Equilibrated monomer Rg averages from oligomers

Oligomeric state	rg1	rg2	rg3	rg_Avg	rg_Std
2	9.73	9.65	9.83	9.74	0.09
3	9.98	10.59	10.05	10.21	0.33
4	10.06	10.64	10.79	10.50	0.39
5	10.33	10.58	10.17	10.36	0.21
6	10.15	10.91	10.48	10.51	0.38
7	10.61	10.19	10.89	10.56	0.35
14	10.55	10.07	10.50	10.37	0.27
30	10.57	10.93	NA*	10.75	0.25

* Only duplicates were performed

Appendix I. Anisotropic Growth Model

In an isotropic growth model, n peptides aggregate to form a spherical shape. However, this idealized growth may not accurately represent cases where deviations from a sphere occur, such as the formation of an ellipsoidal shape or a rugged surface. To address this, we introduce an anisotropic growth model incorporating a distortion factor, σ, which quantifies the degree of deviation from a sphere. As illustrated in the graph below, higher σ values indicate a more pronounced ellipsoidal aggregation.

For a convex object, its averaged shadow area is one-quarter of its total surface area, as proved by Augustin-Louis Cauchy in his thesis:

$$\mathrm{E}[A_{\text{shadow}}] = \frac{1}{4}S \qquad (a1)$$

where $\mathrm{E}[A_{\text{shadow}}]$ is the expected area value of the shadow, S is the area value of the object surface.

The approximate surface area of an ellipsoid is given by:

$$S \approx 4\pi\left(\frac{a^p b^p + a^p c^p + b^p c^p}{3}\right)^{\frac{1}{p}} \qquad (a2)$$

where $a,\ b,\ c$ are the semi-axes of the ellipsoid, and p = 1.6075.

Let CCS be the expected value of shadow, and let $b = ia,\ c = ja$:

$$\mathrm{CCS} = \frac{S}{4} = \pi\left(\frac{i^p + j^p + i^p j^p}{3}\right)^{\frac{1}{p}} a^2 \qquad (a3)$$

For a small and a large ellipsoid:

$$V_0 = \frac{4}{3}\pi a_0^3 i_0 j_0 \qquad (a4), \qquad\qquad V = \frac{4}{3}\pi a^3 ij \qquad (a5)$$

Assume n small ellipsoids with individual volume V_0 are packed into a larger ellipsoid with volume V :

$$nV_0 = V \qquad (a6)$$

Substituting the volumes of the small and large ellipsoids:

$$n \cdot \frac{4}{3}\pi a_0^3 i_0 j_0 = \frac{4}{3}\pi a^3 ij \qquad (a7)$$

Canceling constants and solving for a , the semi-major axis of the large ellipsoid:

$$a = \left(\frac{n \cdot i_0 j_0}{ij}\right)^{\frac{1}{3}} a_0 \qquad (a8)$$

Substituting equation a8 to a3:

$$\text{CCS} = \pi\left(\frac{i^p + j^p + i^p j^p}{3}\right)^{\frac{1}{p}} \cdot \left(\frac{n i_0 j_0}{ij}\right)^{\frac{2}{3}} a_0^2 \qquad (a9)$$

Similar to equation a3, for a small ellipsoid:

$$\Omega_0 = \pi\left(\frac{i_0^p + j_0^p + i_0^p j_0^p}{3}\right)^{\frac{1}{p}} a_0^2 \qquad (a10)$$

Rearrange equation a10 and substitute a_0^2 back into equation a9:

$$\text{CCS} = \left(\frac{i_0 j_0}{ij}\right)^{\frac{2}{3}} \cdot \frac{(i^p + j^p + i^p j^p)^{\frac{1}{p}}}{\left(i_0^p + j_0^p + i_0^p j_0^p\right)^{\frac{1}{p}}} \cdot \Omega_0 \cdot n^{\frac{2}{3}} \qquad (a11)$$

Define the first two terms as a distortion factor $\boldsymbol{\sigma}$, the final simplified form of the CCS is:

$$\text{CCS} = \Omega = \sigma \cdot \Omega_0 \cdot n^{\frac{2}{3}} \qquad (a12)$$

The sigma value and matching eccentricity range

Eccentricity is a well-defined concept for ellipses but not for general ellipsoids. Similar to the definition of eccentricity for an ellipse, $e = \sqrt{1 - \frac{b^2}{a^2}}$, we define the eccentricity for an ellipsoid as equation b1, where the numerator is the square of the smallest axis, and the denominator is the square of the largest axis. Deriving an analytical solution that directly relates σ (the distortion factor, equation b2) with e is complex, but numerical solutions can be readily simulated. The following table compares σ with e: each σ represents a range of e, with larger σ values corresponding to larger e values within a given range.

$$e = \sqrt{1 - \frac{(\min\{a, b, c\})^2}{(\max\{a, b, c\})^2}} = \sqrt{1 - \frac{(\min\{1, i, j\})^2}{(\max\{1, i, j\})^2}} \tag{b1}$$

$$\sigma = \left(\frac{i_0 j_0}{ij}\right)^{\frac{2}{3}} \cdot \frac{(i^p + j^p + i^p j^p)^{\frac{1}{p}}}{\left(i_0^p + j_0^p + i_0^p j_0^p\right)^{\frac{1}{p}}} \tag{b2}$$

From the numerical simulation results, the e value ranges are not linearly proportional to σ values. When σ approaches 1, small changes in σ result in more significant changes in the e value. Nevertheless, the trend indicates that increasing σ corresponds to increasing e values. It is also worth noting that the anisotropic model is based on the assumption of a convex body, meaning the object must have a smooth surface and be strictly convex in mathematical terms. However, in real experiments or simulation data, these conditions are often not met, leading to larger σ values than those predicted by the theoretical model.

Table S4. Sigma value and matching eccentricity range.

σ	e_{min}	e_{max}
1.00	0.0424*	0.1926
1.01	0.6088	0.6495
1.02	0.6920	0.7346
1.03	0.7398	0.7834
1.04	0.7727	0.8167
1.05	0.7978	0.8414
1.10	0.8690	0.9092
1.15	0.9042	0.9405
1.20	0.9256	0.9584
1.25	0.9402	0.9697
1.30	0.9512	0.9772
1.40	0.9646	0.9864
1.50	0.9734	0.9914

* The e_{min} should ideally be 0.00. However, due to the limitation of numerical simulation precision, this value cannot be achieved unless the precision is set extremely high.

Appendix II: Fuzzy Oil Drop Model

This directional hydrophobicity model is constructed in three stages: First, the entire molecule is aligned to its principal axes via PCA so that its longest geometrical feature (the greatest variance direction) becomes the z-axis, with the two shorter axes as y and x. Second, for each residue and each Cartesian direction (x, y, z), two key statistical descriptors are computed: the mean position (representing the residue's center along the axis) and the spreadness, defined as the root-mean-square deviation (RMSD) of all atoms within the residue around its mean. The spreadness reflects how spatially extended or compact the residue is along that specific direction. Third, each residue's contribution to the hydrophobicity profile is modeled as a Gaussian function. The Gaussian is centered at the normalized mean position and has a standard deviation equal to the residue's spreadness. Prior to this projection, each spatial direction is rescaled to the normalized interval $[-1,1]$ so bins are comparable across directions.

By implementing a predefined hydrophobicity scale, each residue is assigned to a distinct hydrophobicity value, such that more hydrophobic residues yield higher Gaussian amplitudes, while less hydrophobic or hydrophilic residues produce flatter or even inverted Gaussians. Combined with the direction-specific spreadness, this Gaussian mapping enables a smooth spatial distribution of hydrophobicity. Residues with larger spatial extent contribute more broadly to the profile, reflecting their physical size and conformational flexibility, whereas more compact residues result in narrower peaks. This approach effectively integrates both the magnitude and spatial footprint of each residue's hydrophobic character into a continuous, directionally resolved profile. Summing these Gaussian contributions over all residues produces a hydrophobicity profile that encodes both the magnitude and spatial extent of residue properties. Because residues are not point-like in reality, incorporating spreadness and using Gaussian functions help to accurately capture the three-dimensional nature of hydrophobicity, thereby avoiding unrealistic, singular spikes and better approximating hydrophobicity distributions.

Python Implementation:

```python
import numpy as np
import matplotlib.pyplot as plt
import mdtraj as md
from scipy.stats import norm
import matplotlib.ticker as ticker
import os

# Font configuration
plt.rc('font', family='Liberation Sans', size=24)

# ===============================
# User Options
# ===============================
use_pca = True
view_directions = ['x', 'y', 'z']
average_over_all_directions = False
pdb_file = '<your_pdb_file>.pdb'
figure_name = '<output_path>/fuzzy_oil_drop.png'
pdb_output = '<output_path>/'

# ===============================
# Load and Align Structure
# ===============================
traj = md.load(pdb_file)
coords = traj.xyz[0]

if use_pca:
    coords_centered = coords - np.mean(coords, axis=0)
    cov = np.cov(coords_centered.T)
    eigvals, eigvecs = np.linalg.eigh(cov)
    eigvecs = eigvecs[:, np.argsort(eigvals)[::-1]]
    rotation_matrix = np.vstack([eigvecs[:, 2], eigvecs[:, 1], eigvecs[:, 0]]).T
    traj.xyz[0] = coords_centered @ rotation_matrix
    rotated_name = os.path.splitext(os.path.basename(pdb_file))[0] + '_rotated.pdb'
    traj.save(os.path.join(pdb_output, rotated_name))

# ===============================
# Hydrophobicity Scale
# ===============================
hydrophobicity_scale = {
    'ALA': 1.8, 'ARG': -4.5, 'ASN': -3.5, 'ASP': -3.5, 'CYS': 2.5, 'GLN': -3.5, 'GLU': -3.5,
    'GLY': -0.4, 'HIS': -3.2, 'ILE': 4.5, 'LEU': 3.8, 'LYS': -3.9, 'MET': 1.9, 'PHE': 2.8,
    'PRO': -1.6, 'SER': -0.8, 'THR': -0.7, 'TRP': -0.9, 'TYR': -1.3, 'VAL': 4.2,
    'D6M': 4.5
}
max_h = max(abs(v) for v in hydrophobicity_scale.values())

def get_hydrophobicity(resname):
    return hydrophobicity_scale.get(resname, 0) / max_h

def calc_spread(positions):
    center = np.mean(positions, axis=0)
    return np.sqrt(np.mean((positions - center) ** 2, axis=0))

# ===============================
# Analysis
# ===============================
line_styles = {'x': '--', 'y': '--', 'z': '--'}
colors = ['red', 'green', 'blue']
alpha = 0.55
num_bins = 100
bin_edges = np.linspace(-1, 1, num_bins + 1)
```

```python
bin_centers = 0.5 * (bin_edges[:-1] + bin_edges[1:])
profiles, used_dirs = [], []

dirs_to_process = ['x', 'y', 'z'] if average_over_all_directions else view_directions

for i, direction in enumerate(view_directions):
    axis = {'x': 0, 'y': 1, 'z': 2}[direction]
    direction_vals = traj.xyz[0][:, axis]
    norm_vals = 2 * (direction_vals - np.min(direction_vals)) / (np.max(direction_vals) - np.min(direction_vals)) - 1

    profile = np.zeros(num_bins)
    for res in traj.topology.residues:
        h = get_hydrophobicity(res.name)
        atom_idx = [atom.index for atom in res.atoms]
        coords = traj.xyz[0][atom_idx]
        spread = calc_spread(coords)[axis]
        center = np.mean(coords[:, axis])
        norm_center = 2 * (center - np.min(direction_vals)) / (np.max(direction_vals) - np.min(direction_vals)) - 1
        idx_range = np.digitize([norm_center - 3 * spread, norm_center + 3 * spread], bin_edges) - 1
        idx_range = np.clip(idx_range, 0, num_bins - 1)
        spread_range = np.arange(idx_range[0], idx_range[1] + 1)
        profile[spread_range] += h * norm.pdf(bin_centers[spread_range], loc=norm_center, scale=spread)

    profiles.append(profile)
    used_dirs.append(direction)

# ===============================
# Averaging if Required
# ===============================
profiles = np.array(profiles)
if average_over_all_directions and set(used_dirs) != set(['x', 'y', 'z']):
    for direction in set(['x', 'y', 'z']) - set(used_dirs):
        # Repeat analysis for missing directions (same logic)
        pass  # Already covered if `view_directions` = ['x', 'y', 'z']

avg_profile = np.mean(profiles, axis=0)
std_profile = np.std(profiles, axis=0)

# ===============================
# Plotting
# ===============================
plt.figure(figsize=(15, 5))
for i, direction in enumerate(view_directions):
    if direction in used_dirs:
        plt.plot(bin_centers, profiles[i], label=f'{direction} direction',
                 linestyle=line_styles[direction], color=colors[i], alpha=alpha, lw=3)

plt.plot(bin_centers, avg_profile, label='Average', color='black', lw=4.5)
plt.fill_between(bin_centers, avg_profile - std_profile, avg_profile + std_profile,
                 color='black', alpha=0.15, label='Std Dev')

plt.xlabel('Normalized Position')
plt.ylabel('Hydrophobicity')

ax = plt.gca()
ax.yaxis.set_major_locator(ticker.MultipleLocator(20))
ax.yaxis.set_minor_locator(ticker.AutoMinorLocator(2))

ax.tick_params(axis='y', which='major', width=2.5, length=9)
ax.tick_params(axis='y', which='minor', width=1.5, length=5)
ax.tick_params(axis='x', which='major', width=2.0, length=7)
```

```
ax.tick_params(axis='x', which='minor', width=1.2, length=4)

plt.tight_layout()
plt.savefig(figure_name, dpi=300, transparent=True)
plt.show()

```

Appendix III. Free Energy Landscape

Free energy landscape refers to the mapping of the system's underlying free energy profile onto a reduced set of coordinates, providing a simplified yet informative view of the conformational space. Rather than working directly with high-dimensional atomic coordinates, the landscape profile construction relies on a projection onto collective variables or low-dimensional descriptors that capture the essential motions or features of interest. These coordinates can be principal components derived from PCA[13], distances between key atoms or domains[14], dihedral angles[15], or other structural metrics.[16] By estimating the probability density of sampled configurations in this reduced space and applying the Boltzmann equation (equation c1), the free energy landscape can be constructed.

$$F(x, y) = -kT \ln P\,(x, y) \qquad (c1)$$

, where F(x, y) is the free energy at point (x, y), P(x, y) is estimated probability density at that point, k is the Boltzmann constant, and T is the absolute temperature.

A central challenge in constructing reliable free energy surfaces is how to estimate the probability distribution without introducing artifacts from data binning or imbalances in sampling. Histogram-based approaches are sensitive to the choice of bin size and can suffer from over-smoothing or noise amplification, especially when regions of the landscape are either oversampled or sparsely visited during simulation.

To address the limitations of histogram-based approaches, the method adopts a statistically principled and adaptive strategy. Instead of binning, it applies a bivariate kernel density estimation (KDE) with adaptive bandwidth selection using the normal reference rule. This procedure fits a smooth, continuous probability distribution to the projected data, avoiding rigid discretization and bin-edge artifacts. The estimated density is given by:

$$\hat{P}(x, y) = \frac{1}{n h_x h_y} \sum_{i=1}^{n} K\left(\frac{x - x_i}{h_x}, \frac{y - y_i}{h_y}\right) \qquad (c2)$$

, where $\hat{P}(x, y)$ is estimated probability density at point (x, y), n is the total number of data points, (x_i, y_i) are the coordinates of the i-th data point, (h_x, h_y) are automatically chosen kernel bandwidths along the x and y axes, and K represnts a kernel function (Gaussian function).

This approach smooths the density based on the data distribution itself, eliminating the need for arbitrary binning and reducing discontinuities caused by fixed grid edges. A regular evaluation grid is then defined, not for binning raw data, but to consistently evaluate the continuous KDE. This ensures standardized visual and computational comparisons across datasets.

To further guard against over- or under-sampling artifacts, a bootstrap procedure is implemented to quantify statistical variability in the density at each grid point. The standard deviation of bootstrapped KDE values is used to define a noise floor. A minimum threshold is then imposed on the estimated density to prevent singularities in the subsequent free energy transformation. This adaptive thresholding prevents unreliable estimates in sparsely sampled regions from dominating the free energy landscape, ensuring numerical stability while retaining meaningful features. The resulting free energy surface is then refined using total variation (TV) denoising, which filters out non-physical, high-frequency fluctuations while preserving sharp features such as energy wells and barriers. The isotropic TV norm is defined as:

$$TV(\mathrm{f}) = \sum_{i,j} \sqrt{\left(f_{i+1,j} - f_{i,j}\right)^2 + \left(f_{i,j+1} - f_{i,j}\right)^2} \qquad (c3)$$

, where TV(f) is the total variation of the free energy, $f_{i,j}$ is the free energy at a grid point (i, j).

The optimal regularization weight is automatically determined by identifying the point of maximum curvature on the L-curve, balancing smoothness with fidelity to the original data.To assess uncertainty and identify robust features, the entire free energy transformation is repeated across all bootstrapped density samples. This provides a map of the standard deviation in free energy values at each location.

Local minima are identified by locating points lower than their immediate neighbors, with edge regions excluded to avoid boundary artifacts. Minima are further filtered by pairwise distance and relative energy to remove redundant or spurious entries. The final set of minima reflects physically meaningful conformational states. These minima are highlighted on the three-dimensional energy landscape and annotated with both their free energy values and optionally associated uncertainties. Each minimum is then traced back to its corresponding structure in the original dataset, enabling downstream structural interpretation and clustering for further analysis.

Python Implementation:

```
def plot_energy_3d_with_smoothing(projected_data_1,
                                  projected_data_2,
                                  kT=0.596,
                                  file_name=None,
                                  n_boot=50,
                                  bootstrap_sigma_factor=3):
    # --- flatten & mask NaNs ---
    x = projected_data_1.flatten()
    y = projected_data_2.flatten()
    mask = ~np.isnan(x) & ~np.isnan(y)
    x, y = x[mask], y[mask]
    n_points = x.size
    print(f"Number of data points: {n_points}")
    data = np.vstack([x, y])

    # --- adaptive KDE (normal_reference) ---
    kde = KDEMultivariate(data.T,
                          var_type='cc',
                          bw='normal_reference')

    # --- build evaluation grid ---
    grid_resolution = 100
    xi = np.linspace(x.min(), x.max(), grid_resolution)
    yi = np.linspace(y.min(), y.max(), grid_resolution)
    X_new, Y_new = np.meshgrid(xi, yi)
    grid_coords = np.vstack([X_new.ravel(), Y_new.ravel()])

    # --- evaluate density on grid ---
    density = kde.pdf(grid_coords.T).reshape(X_new.shape)

    # --- bootstrap to estimate density noise floor ---
    print("Running bootstrap to estimate density noise floor...")
    dens_boot = np.zeros((n_boot, X_new.size))
    for b in range(n_boot):
        idx_bs = np.random.choice(n_points, n_points, replace=True)
        data_bs = data[:, idx_bs]
        kde_bs = KDEMultivariate(data_bs.T,
                                 var_type='cc',
                                 bw='normal_reference')
        dens_boot[b, :] = kde_bs.pdf(grid_coords.T)
    std_density = np.std(dens_boot, axis=0).reshape(X_new.shape)
    # set floor at, e.g., 3σ of the bootstrapped density noise:
    sigma_floor = np.percentile(std_density, 0.1) # 0.1th percentile
    floor_val   = sigma_floor * bootstrap_sigma_factor
    print(f"Density noise floor: {floor_val:.4g}")
    density = np.maximum(density, floor_val)

    # --- convert to free energy ---
    density[density <= 0] = floor_val  # safety
    free_energy = -kT * np.log(density)
    free_energy -= np.nanmax(free_energy)

    # --- pad for TV denoising ---
    pad = 10
    u = np.pad(free_energy, pad_width=pad, mode='wrap')

    # --- auto eps based on data scale ---
    data_range = u.max() - u.min()
    print(f"Data range: {data_range:.4g}")
    eps_auto = 1e-2 * data_range
    # 1e-2 is stable for current settings
    print(f"Auto TV eps: {eps_auto:.4g}")
```

```

    # --- automatic TV weight via L-curve corner ---
    weights = np.logspace(-10, 10, 50)
    phis, tvs, solutions = [], [], []
    for w in weights:
        v = denoise_tv_bregman(u,
                               weight=w,
                               eps=eps_auto,
                               isotropic=True)
        solutions.append(v)
        phis.append(np.linalg.norm(v - u)**2)
        # discrete isotropic TV on interior:
        g0 = v[1:, :-1] - v[:-1, :-1]
        g1 = v[:-1, 1:] - v[:-1, :-1]
        tvs.append(np.sum(np.sqrt(g0**2 + g1**2)))
    log_phi = np.log(phis)
    log_tv  = np.log(tvs)
    curv    = _compute_curvature(log_tv, log_phi)
    best_idx = np.argmax(curv)
    best_w   = weights[best_idx]
    print(f"Selected TV weight: {best_w:.4g}")

    # --- extract optimal denoised FES ---
    v_opt    = solutions[best_idx]
    final_fe = v_opt[pad:-pad, pad:-pad]

    # --- propagate bootstrap to free energy uncertainty ---
    print("Computing free-energy bootstrap uncertainties...")
    fe_boot = -kT * np.log(np.maximum(dens_boot, floor_val))
    fe_std_map = fe_boot.std(axis=0).reshape(X_new.shape)

    # --- find local minima ---
    neighborhood = generate_binary_structure(2, 2)
    local_min = minimum_filter(final_fe,
                               footprint=neighborhood) == final_fe
    edge = 2
    local_min[:edge, :] = False
    local_min[-edge:, :] = False
    local_min[:, :edge] = False
    local_min[:, -edge:] = False

    z_min = final_fe[local_min]
    x_min = X_new[local_min]
    y_min = Y_new[local_min]
    z_std_min = fe_std_map[local_min]  # bootstrap std at minima

    # select significant minima
    thr = np.percentile(final_fe, 30)
    keep = z_min <= thr
    x_min, y_min = x_min[keep], y_min[keep]
    z_min, z_std_min = z_min[keep], z_std_min[keep]

    # prune nearby minima
    if len(z_min) > 1:
        pts = np.column_stack((x_min, y_min))
        D   = distance.squareform(distance.pdist(pts))
        to_rm = set()
        for i in range(len(z_min)):
            if i in to_rm: continue
            for j in range(i+1, len(z_min)):
                if D[i, j] < 0.15:
                    to_rm.add(j if z_min[i] < z_min[j] else i)
        keep_idx = [i for i in range(len(z_min)) if i not in to_rm]
        x_min, y_min = x_min[keep_idx], y_min[keep_idx]
        z_min, z_std_min = z_min[keep_idx], z_std_min[keep_idx]
```

```
    # --- plotting ---
    fig = plt.figure(figsize=(10, 8))
    ax  = fig.add_subplot(111, projection='3d')
    cmap = truncate_colormap(plt.get_cmap('coolwarm'), 0.1, 0.9)
    ax.plot_surface(X_new, Y_new, final_fe,
                    cmap=cmap, edgecolor='black', linewidth=0.03, alpha=0.8,
                    antialiased=True, rstride=1, cstride=1)
    ax.view_init(elev=27, azim=-72)
    ax.set_xlabel('Intrinsic Coordinate 1',
                  fontsize=base_fontsize, labelpad=base_fontsize*0.15)
    ax.set_ylabel('Intrinsic Coordinate 2',
                  fontsize=base_fontsize, labelpad=base_fontsize*0.15)
    ax.set_zlabel('Free Energy (kcal/mol)',
                  fontsize=base_fontsize, labelpad=base_fontsize*0.75)
    ax.xaxis.set_label_coords(0.5, -0.8)
    ax.yaxis.set_label_coords(-0.8, 0.5)
    ax.zaxis.set_label_coords(0.5, 1.05)
    ax.xaxis.set_major_locator(plt.MaxNLocator(integer=True))
    ax.yaxis.set_major_locator(plt.MaxNLocator(integer=True))
    ax.xaxis.set_minor_locator(AutoMinorLocator())
    ax.yaxis.set_minor_locator(AutoMinorLocator())
    ax.tick_params(axis='both', which='major', pad=-2, labelsize=base_fontsize*0.9)
    ax.tick_params(axis='z', which='major', pad=8, labelsize=base_fontsize*0.9)
    for axis in (ax.xaxis, ax.yaxis, ax.zaxis):
        axinfo = axis._axinfo['tick']  # grab the dict controlling the 3D ticks
        axinfo['inward_factor']  = 0.2  # how far tick goes “in” toward plot
        axinfo['outward_factor'] = 0.1  # how far tick goes “out” from plot

    for pane in (ax.xaxis.pane, ax.yaxis.pane, ax.zaxis.pane):
        pane.set_facecolor((1,1,1,0.0))
    fig.set_facecolor('none')
    ax.set_facecolor('none')
    ax.grid(False)

    ax.scatter(x_min, y_min, z_min + 1.1,
               color='orange', marker='*', s=100, alpha=0.9,
               label='Local Minima')
    for xi_, yi_, zi_, zs_ in zip(x_min, y_min, z_min, z_std_min):
        ax.text(xi_, yi_, zi_ + 2.2, f"{zi_:.2f}", # Or {zi_:.2f}±{zs_:.2f}
                color='black', weight='bold',
                ha='center', va='bottom',
                fontsize=base_fontsize * 0.75)
    ax.legend(
    fontsize=base_fontsize * 0.85,
    loc='upper right',
    bbox_to_anchor=(0.88, 0.78),   # xy position in 0-1 range
    borderaxespad=0.5          )

    # --- map minima back to original indices & report ---
    samples = np.vstack([projected_data_1.flatten(),
                         projected_data_2.flatten()]).T
    tree = cKDTree(samples)
    data_idx = [tree.query((xi, yi))[1] + 1
                for xi, yi in zip(x_min, y_min)]
    minima_df = pd.DataFrame({
        'data_index':      data_idx,
        'X Coordinate':    x_min,
        'Y Coordinate':    y_min,
        'Free Energy':     z_min,
        'FE StdDev (boot)': z_std_min
    })
    print(minima_df)

    if file_name:
```

```
        plt.savefig(file_name, dpi=600,
                    bbox_inches='tight', pad_inches=0.05,
                    bbox_extra_artists=[
                        ax.xaxis.label,
                        ax.yaxis.label,
                        ax.zaxis.label
                    ], transparent=True)
    plt.show()

    xyz = np.column_stack((X_new.ravel(),
                           Y_new.ravel(),
                           final_fe.ravel()))
    minima_xy = list(zip(x_min, y_min))
    return xyz, X_new, Y_new, final_fe, minima_xy

```

Reference

1. S. J. Marrink, H. J. Risselada, S. Yefimov, D. P. Tieleman and A. H. de Vries, The MARTINI Force Field: Coarse Grained Model for Biomolecular Simulations, *The Journal of Physical Chemistry B*, 2007, **111**, 7812-7824.
2. L. Monticelli, S. K. Kandasamy, X. Periole, R. G. Larson, D. P. Tieleman and S.-J. Marrink, The MARTINI Coarse-Grained Force Field: Extension to Proteins, *Journal of Chemical Theory and Computation*, 2008, **4**, 819-834.
3. J. Huang, S. Rauscher, G. Nawrocki, T. Ran, M. Feig, B. L. de Groot, H. Grubmüller and A. D. MacKerell, CHARMM36m: an improved force field for folded and intrinsically disordered proteins, *Nature Methods*, 2017, **14**, 71-73.
4. M. J. Abraham, T. Murtola, R. Schulz, S. Páll, J. C. Smith, B. Hess and E. Lindahl, GROMACS: High performance molecular simulations through multi-level parallelism from laptops to supercomputers, *SoftwareX*, 2015, **1-2**, 19-25.
5. W. G. Touw, C. Baakman, J. Black, T. A. H. te Beek, E. Krieger, R. P. Joosten and G. Vriend, A series of PDB-related databanks for everyday needs, *Nucleic Acids Research*, 2015, **43**, D364-D368.
6. S. Jo, T. Kim, V. G. Iyer and W. Im, CHARMM-GUI: A web-based graphical user interface for CHARMM, *Journal of Computational Chemistry*, 2008, **29**, 1859-1865.
7. Y. Qi, X. Cheng, W. Han, S. Jo, K. Schulten and W. Im, CHARMM-GUI PACE CG Builder for Solution, Micelle, and Bilayer Coarse-Grained Simulations, *Journal of Chemical Information and Modeling*, 2014, **54**, 1003-1009.
8. T. A. Wassenaar, K. Pluhackova, R. A. Böckmann, S. J. Marrink and D. P. Tieleman, Going Backward: A Flexible Geometric Approach to Reverse Transformation from Coarse Grained to Atomistic Models, *Journal of Chemical Theory and Computation*, 2014, **10**, 676-690.
9. G. Bussi, D. Donadio and M. Parrinello, Canonical sampling through velocity rescaling, *The Journal of Chemical Physics*, 2007, **126**.
10. M. Parrinello and A. Rahman, Polymorphic transitions in single crystals: A new molecular dynamics method, *Journal of Applied Physics*, 1981, **52**, 7182-7190.
11. A. Patriksson and D. van der Spoel, A temperature predictor for parallel tempering simulations, *Physical Chemistry Chemical Physics*, 2008, **10**, 2073-2077.
12. W. G. Touw, C. Baakman, J. Black, T. A. te Beek, E. Krieger, R. P. Joosten and G. Vriend, A series of PDB-related databanks for everyday needs, *Nucleic Acids Res*, 2015, **43**, D364-368.
13. E. Papaleo, P. Mereghetti, P. Fantucci, R. Grandori and L. De Gioia, Free-energy landscape, principal component analysis, and structural clustering to identify representative conformations from molecular dynamics simulations: The myoglobin case, *Journal of Molecular Graphics and Modelling*, 2009, **27**, 889-899.
14. L. Kollias, D. C. Cantu, M. A. Tubbs, R. Rousseau, V.-A. Glezakou and M. Salvalaglio, Molecular Level Understanding of the Free Energy Landscape in Early Stages of Metal–Organic Framework Nucleation, *Journal of the American Chemical Society*, 2019, **141**, 6073-6081.

15. L. Fallon, K. A. A. Belfon, L. Raguette, Y. Wang, D. Stepanenko, A. Cuomo, J. Guerra, S. Budhan, S. Varghese, C. P. Corbo, R. C. Rizzo and C. Simmerling, Free Energy Landscapes from SARS-CoV-2 Spike Glycoprotein Simulations Suggest that RBD Opening Can Be Modulated via Interactions in an Allosteric Pocket, *Journal of the American Chemical Society*, 2021, **143**, 11349-11360.
16. B. Zhang, W. Zheng, G. A. Papoian and P. G. Wolynes, Exploring the Free Energy Landscape of Nucleosomes, *Journal of the American Chemical Society*, 2016, **138**, 8126-8133.
